# Eocene (50–55 Ma) greenhouse climate recorded in nonmarine rocks of San Diego, CA, USA

**DOI:** 10.1038/s41598-024-53210-0

**Published:** 2024-01-31

**Authors:** Adrian P. Broz, Devin Pritchard-Peterson, Diogo Spinola, Sarah Schneider, Gregory Retallack, Lucas C. R. Silva

**Affiliations:** 1 Department of Earth, Atmospheric and Planetary Sciences, Purdue University, Lafayette, IN USA; 2 Department of Earth Sciences, University of Oregon, Eugene, OR USA; 3 Dudek Environmental Consulting, Encinitas, CA USA; 4 Department of Ecosystem Science and Management, University of North British Columbia, Prince George, BC Canada; 5 Environmental Studies Program, University of Oregon, Eugene, OR USA; 6 Department of Biology, University of Oregon, Eugene, OR USA

**Keywords:** Palaeoclimate, Geochemistry, Mineralogy, Sedimentology

## Abstract

Nonmarine rocks in sea cliffs of southern California store a detailed record of weathering under tropical conditions millions of years ago, where today the climate is much drier and cooler. This work examines early Eocene (~ 50–55 million-year-old) deeply weathered paleosols (ancient, buried soils) exposed in marine terraces of northern San Diego County, California, and uses their geochemistry and mineralogy to reconstruct climate and weathering intensity during early Eocene greenhouse climates. These Eocene warm spikes have been modeled as prequels for ongoing anthropogenic global warming driven by a spike in atmospheric CO_2_. Paleocene-Eocene thermal maximum (PETM, ~ 55 Ma) kaolinitic paleosols developed in volcaniclastic conglomerates are evidence of intense weathering (CIA > 98) under warm and wet conditions (mean annual temperature [MAT] of ~ 17 °C  ± 4.4 °C and mean annual precipitation [MAP] of ~ 1500 ± 299 mm). Geologically younger Early Eocene climatic optimum (EECO, 50 Ma) high shrink-swell (Vertisol) paleosols developed in coarse sandstones are also intensely weathered (CIA > 80) with MAT estimates of ~ 20 °C ± 4.4 °C but have lower estimated MAP (~ 1100 ± 299 mm), suggesting a less humid climate for the EECO greenhouse spike than for the earlier PETM greenhouse spike.

## Introduction

Periods of accentuated greenhouse conditions, characterized by spikes (or excursions) in CO_2_ concentrations exceeding ~ 2000 ppm, punctuated the Earth´s climate during the Paleogene, from the late Paleocene to the Early Eocene (60 to 52 million years ago)^1^. During these epochs, global temperatures often reached more than ten degrees Celsius higher than those of the pre-industrial period^2^. These Eocene CO_2_-driven warm spikes have been modeled as prequels for ongoing anthropogenic global warming^3,4^.

Paleosols (fossil soils) from the Late Paleocene to Eocene epochs have been reported worldwide in Antarctica^5^, Argentina^6^, Australia^7^ and across the United States^8–11^. These paleosols demonstrate markedly more intense weathering conditions than in the same area today and are evidence of the warmer climates that prevailed during the early Cenozoic^6^. Evidence of increased weathering intensity across latitudes is from the formation of deep (~ 30 m) weathering profiles^12^, elevated alteration indices^13^ and abundant kaolinite^14^ which are characteristics of deeply weathered modern soils at present-day equatorial to subequatorial latitudes^15^. The increased weathering intensity on land surfaces during these periods is a direct function of climate and is also influenced by other processes such as vegetation and microbial activity^16^.

New evidence of these warming periods can be seen in a sequence of Eocene paleosols located in today's coastal deserts of southern California, revealing a significantly warmer and wetter paleoclimate relative to the modern arid climate. Early Eocene paleosols in the coastal plains of northwestern Baja California and southwestern California show the effects of intense weathering under a subtropical humid climate^12^. This is consistent with the global greenhouse climates during the Paleocene-Eocene Thermal Maximum (PETM, ~ 55 Ma)^3,10^. An additional global warming event, known as the Early Eocene Climatic Optimum (EECO, 52–50 Ma), also fostered intense weathering in warm, wet climates^17,18^.

Although Cenozoic paleosols of San Diego have been known for several decades^12^, they are now able to be thoroughly examined using a comprehensive set of climofunctions and other quantitative proxies for soil formation conditions^19–22^. Application of these techniques to a new set of deeply weathered Eocene paleosols advances our understanding of how Eocene climate excursions affected land surfaces across latitudes. This work uses the morphology, mineralogy and geochemistry of San Diego paleosols to provide a quantitative assessment of climate and weathering intensity on land during and after Eocene greenhouse spikes.

This work represents the first reconstruction of PETM and EECO precipitation and temperature from a nonmarine site in present-day California.

## Geological setting and Cenozoic greenhouse climate

**Figure 1:**
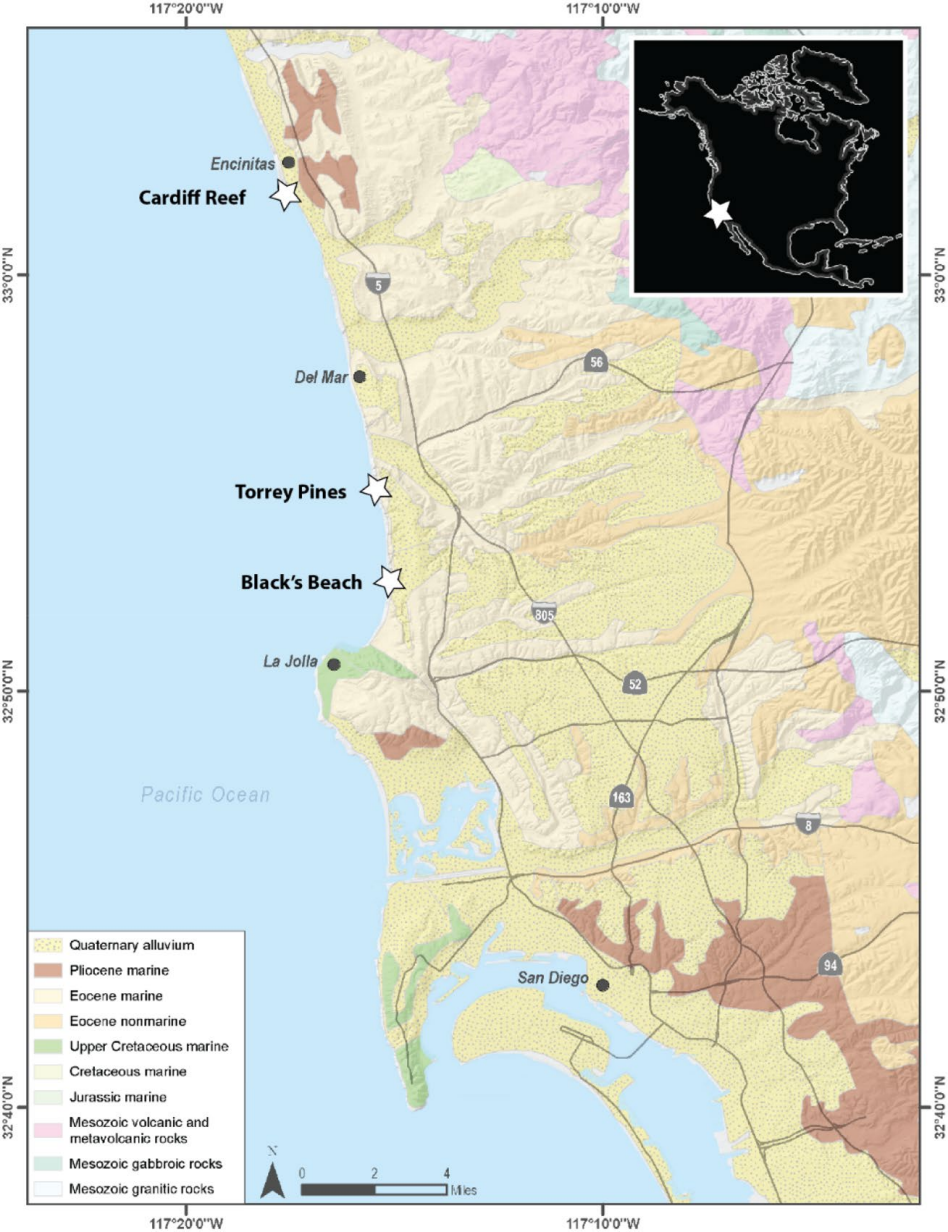
Field areas in northern San Diego County, California, USA. Map was created using ArcGIS Pro 3.1 (https://www.esri.com).

The study area lies within the peninsular Ranges of southern California and is composed primarily of Jurassic to Cretaceous igneous and metamorphic rocks, and Eocene marine and nonmarine sedimentary rocks (Fig. 1). Erosion following mountain-building in the mid-Cretaceous led to the formation of a stable, flat-lying coastal-plain basement that ranges in age from late Cretaceous to early Holocene. This work focuses on two of the coastal plain stratigraphic units of late Cretaceous and early Eocene age that have preserved evidence of intense subaerial alteration.

Shortly after the late Jurassic to mid-Cretaceous Nevadan orogeny, San Diego County was transformed into a low-lying coastal plain that accumulated Cretaceous to Cenozoic nonmarine and marine sedimentary deposits^23,24^. Paleosols of the greater San Diego area developed on Jurassic andesite and andesitic breccia, Rancho Delicias granodiorite, as well as early Eocene [55 Ma] volcanic and volcaniclastic conglomerates of the Mt. Soledad Formation^12^. The discontinuous sequence of weathered intervals begins with Paleocene (~ 56 Ma) kaolinitic Oxisol paleosols at Rancho Delicias, Tijuana, which are nearly 30 m in vertical thickness^12,25^. Approximately 60 km to the north, outcrops of lower Eocene (55 Ma) kaolinitic paleosols of the Mt. Soledad conglomerate are exposed in beach cliffs at Black’s Beach, La Jolla, below Ardath Shale with mollusks of the *Turritella uvasana* zone^24,26^. These are overlain by Middle Eocene (50 Ma) smectite-rich paleosols of the marginal marine Delmar Formation at San Elijo Beach, Cardiff, CA. Paleosols of the Delmar Formation are overlain by late Eocene (~ 40 Ma) Aridisol paleosols of the Friars formation that contain abundant pedogenic carbonate nodules and a variety of vertebrate fossils of the Uintan North American Land mammal Age^27^. This study focused on paleosols of the early Eocene (55 Ma) Mt. Soledad Formation and later early Eocene (50 Ma) Delmar Formation. Paleomagnetic evidence locates southern California at latitudes 35–40° N during the Paleocene and Early Eocene (Smith and Briden, 1977), at least 400 km north of its current latitude of 32^o^ N.

### Mount Soledad formation conglomerate

Conglomerates of the basal Mount Soledad Formation are overlain by the early Middle Eocene Ardath Shale^26^. The Mt. Soledad Formation is a framework-supported, amalgamated conglomerate with exotic clast composition^28^. The composition of the clasts is dominated by quartz phenocryst-bearing rhyolites that originated from present-day Sonoran Desert of Mexico, as well as quartzite and silicified tuff^29^. Conglomerate clasts include approximately 40% rhyolite, 26% black dacites, 13% Santiago Peak Volcanics, 12% schist, 4% plutonic, and 2% intraformational^24^. Paleohydrological reconstruction of the area suggested a 300-km long river with a channel width of 20–80 m and a peak 100-year flood discharge of 30,000 m^3^ S^−1^^12^.

### Delmar formation sandstone

The Eocene (50 Ma) Delmar Formation consists of coarse-grained quartzofeldspathic sandstone that was deposited in shallow marine, intertidal and supratidal facies of the Eocene San Diego Embayment^12^, and is approximately equivalent in age to the Green River Formation in Wyoming^30^. Tidally influenced sedimentary features include an assemblage of largely shallow marine oysters, flaser bedding, inclined cross bedding, interlaminated siltstone and mudstone that follow basal and lateral accretionary surfaces of tidal channels, and local flood and return-surge deposits^24^. Fossil plants such as giant leather fern (*Acrostichum aureum*) also suggest mangrove habitats^31^.

## Materials and methods

### Sample collection and morphological assessment

**Figure 2:**
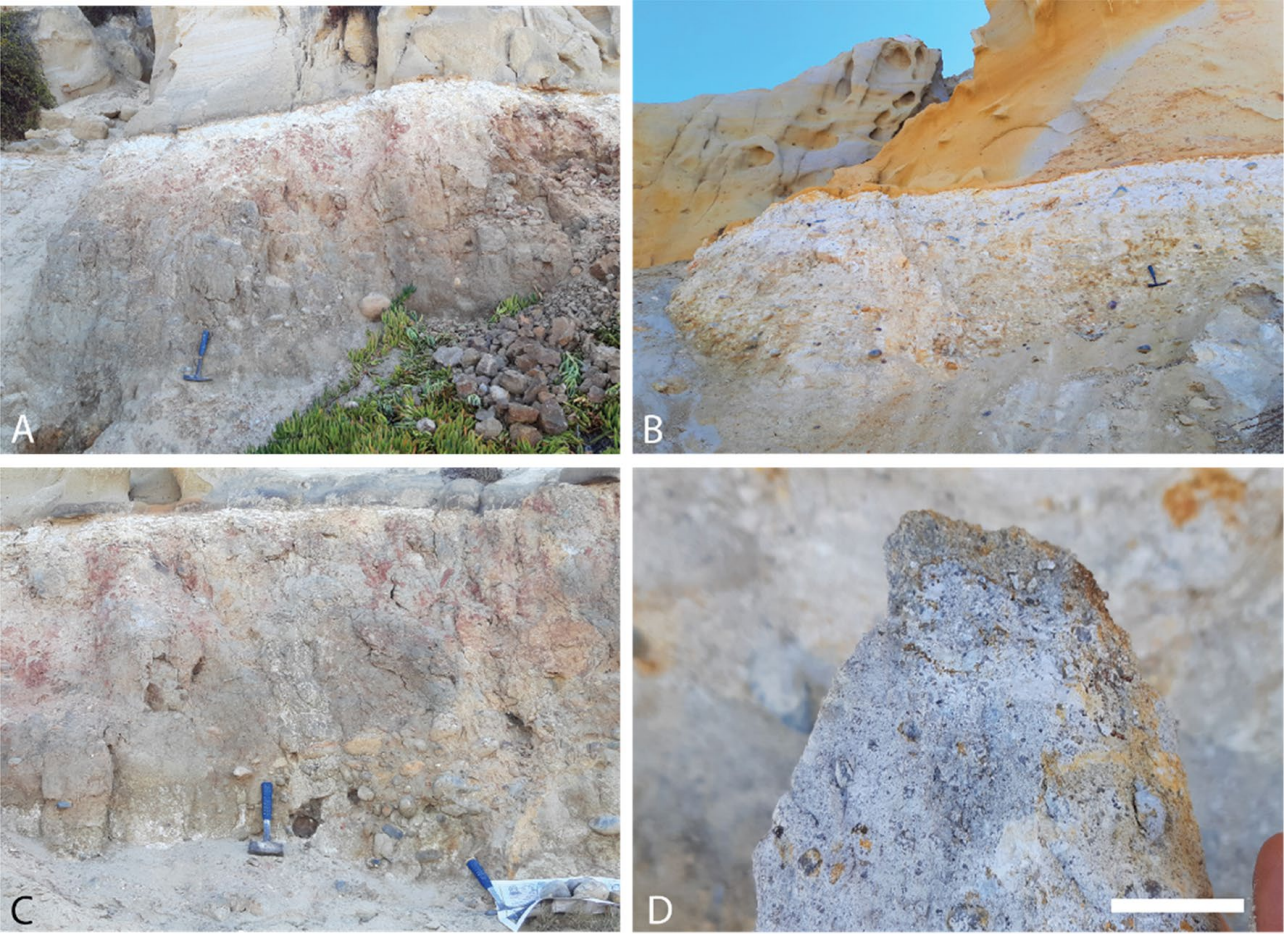
A catena of two severely weathered early Eocene (55 Ma) kaolinitic paleosols in marine terrace at Black’s Beach, La Jolla, California USA (32.895500, − 117.253520). (**A**) White/brown kaolinitic paleosol profile formed in conglomerate of the Eocene (55 Ma) Mt. Soledad Formation and buried by overlying Torrey Formation sandstone (32.89400, − 117.253520); (**B**) Gray/white kaolinitic profile (along strike) also formed in conglomerates of the Eocene (55 Ma) Mt. Soledad Formation and buried by overlying Torrey Formation sandstone; (**C**) large > 10 cm conglomerate clasts in the C-horizon of the brown/white profile; (**D**) hand sample from the A-horizon of gray/white profile showing kaolinite (white) and residual coarse quartz clasts. Scale bar in D) is 2 cm.

**Figure 3:**
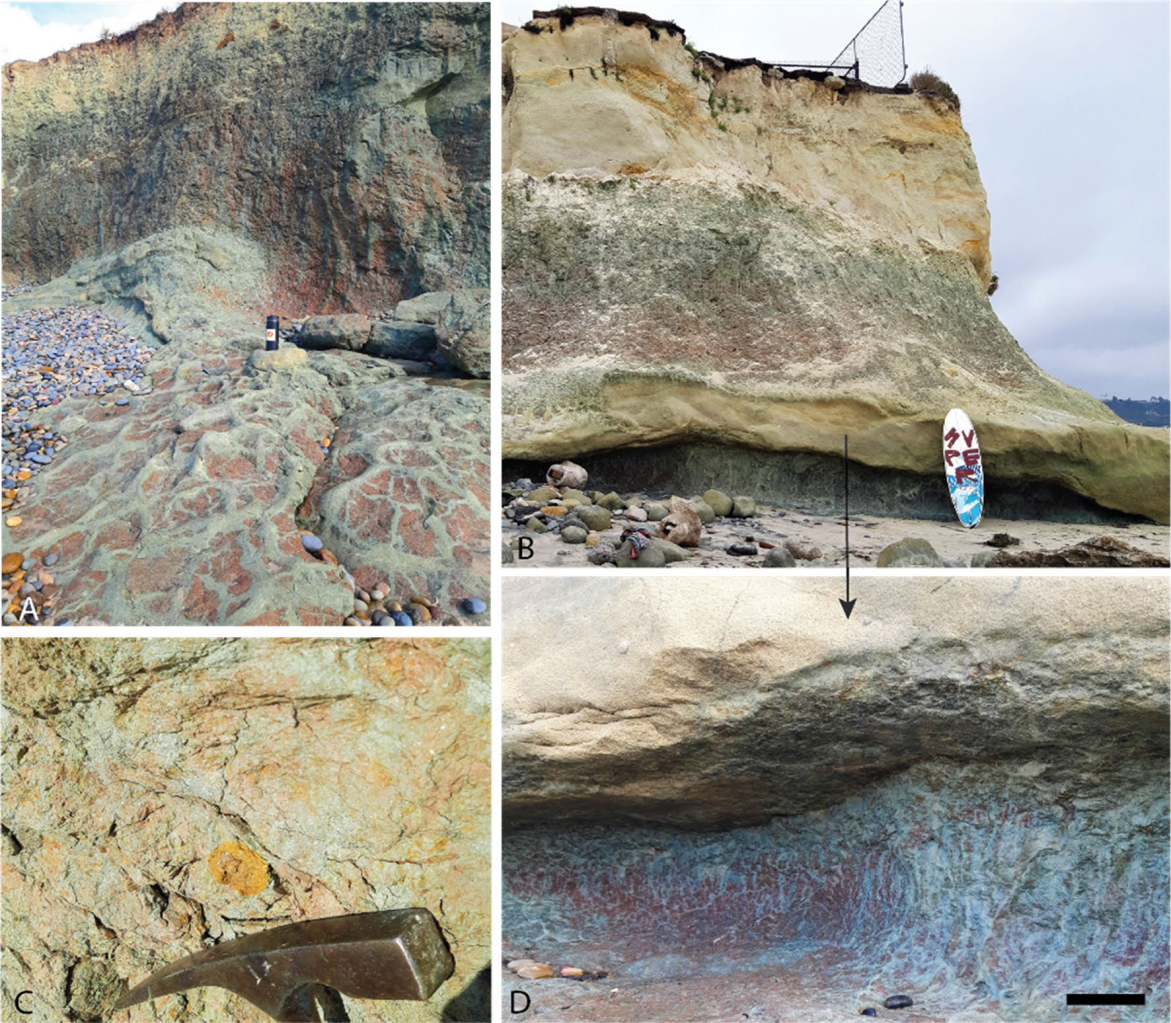
A sequence of Eocene (50 Ma) red clay Vertisol (shrink-swell) paleosols at San Elijo Beach, Cardiff-by-the-sea, California, USA (32.895500, − 117.25352). (**A**) Three successive Vertisol paleosols exposed in marine terraces with soil structures exposed in the shore platform including coarse sand-filled mudcracks; (**C**) common Fe oxide concretions up to 5 cm in diameter in the B-horizons of the lowermost two profiles; (**D**) Drab green/gray coarse sand-filled mudcracks and brick-red matrix of the basal paleosol profile with extensive mottling extending to sea in the shore platform. Scale bar in C) is 10 cm.

Field descriptions and collection of hand samples was performed at Black’s Beach, La Jolla, CA, USA (32.895500, − 117.253520) and at San Elijo Beach, Cardiff-by-the-Sea, CA (32.895500, − 117.25352) (Figs. 2, 3). Five paleosol profiles were sampled. These included a paleocatena of two profiles (approximately 200 m apart) at La Jolla, and a vertical sequence of three successive profiles in Cardiff (“Cardiff Reef”, Fig. S1). Additional profiles of putative mangrove paleosols in supratidal facies of the Delmar Formation were observed at Torrey Pines, CA and descriptions are included in Supplementary information (Figs. S2–S3). The grey paleosols with carbonaceous root traces and oysters at Torrey Pines were not chemically analyzed, because unlike thick red paleosols, they are not developed enough to reveal paleoclimate or other soil forming factors^20^. Hand samples were collected by trenching to approximately 30 cm into the paleosol outcrop for fresh samples. Large, lithified blocks were collected at approximately 20 cm intervals, similar to sampling the horizons of a modern soil profile. The morphology, qualitative grain size, Munsell color and calcareousness of samples were described during collection. Paleosol taxonomic assessment followed the methods and nomenclature of U.S. Soil Taxonony (Soil Survey Staff, 2014). Pedotypes followed the nomenclature of the local Kumeyaay language spoken by the 12 federally-recognized tribes of the region (Field, 2012). A stratigraphic section along depositional strike proposed by^24^ is included in Supplementary Information (Fig. S4).

### Bulk geochemistry

Major element chemistry of paleosols was determined by X-ray fluorescence (XRF) and Pratt titration for FeO at ALS Laboratories, Vancouver, British Columbia (Table S1). Errors for XRF detection of individual elements (Table S1) were calculated from ten replicate measurements of the standard CANMET SDMS2 (British Columbia granodioritic sand). These data were used to calculate molar weathering ratios, indices of alteration, and geochemical mass balance (tau, strain) of each paleosol profile. Bulk density was measured on lithified clasts using the paraffin-clod method^32,33^.These values are provided in Tables S1 and S2.

### Geochemical proxies

The degree of weathering of paleosols can be estimated using molecular ratios as indicators of the soil-forming processes^34^ including salinization (Na_2_O/K_2_O), calcification (CaO + MgO/Al_2_O_3_), clayeyness (Al2O_3_/SiO_2_), base loss (AL_2_O_3_/ CaO + MgO + Na_2_O + K_2_O) and gleization (FeO/Fe_2_O_3_). Salinization is a measure of the salt accumulation in paleosols whereas calcification estimates the accumulation of pedogenic carbonates at depth. Clayeyness and base loss evaluate the extent of hydrolytic weathering and leaching of cations as a function of depth in the profile. Gleization constrains the redox state of the soil before burial, with values > 1 suggesting waterlogged and reducing conditions and values < 1 suggesting well-drained, oxidizing conditions before burial^34,35^. Oxide weight percentages were also used to estimate mean annual precipitation using the CIA-K (chemical index of alteration minus K_2_O) paleoprecipitation proxy^19^, defined as 221.12e^0^^.0197(CIA-K)^ with R^2^ 0.72 and root mean square prediction error of 299 mm^22^, and the CALMAG weathering index, designed for use with Vertisol paleosols^21^, defined as Al_2_O_3_/(Al_2_O_3_ + CaO + MgO) × 100. The CALMAG paleoprecipitation proxy (y = 22.69x – 435.8, R^2^ = 0.90; s.e. ± 108 mm, where x = CALMAG weathering index) was compared with CIA-K paleoprecipitation estimates. We also used recursive partitioning via random forests to estimate paleoprecipitation and prediction error using paleosol geochemistry (RF-MAP 1.0)^22^. Calculated values are provided in Table S3.

### Total inorganic/organic carbon and pH

The pH and total organic carbon (TOC) of samples was assessed to constrain the organic content and diagenetic history of paleosols. Since waterlogged soils can be sites of enhanced organic preservation, especially those with FeO/Fe2O3 < 1^35^, we used elemental analysis to quantify the paleosol organic carbon pool. However, paleosols often contain both ancient and modern carbon as inferred from radiocarbon dating, and distinguishing between the two can be challenging^36^. Furthermore, reconstructing soil pH from paleosols is difficult because diagenesis (e.g., groundwater alteration) can obscure or overprint original soil pH^37^. Paleosol samples were manually encapsulated in 5 × 8 mm tin capsules (sample size approximately (25–70 mg) prior to elemental analysis. Total organic carbon was determined by elemental analysis on a Costech ECS 4010 instrument at the University of Oregon’s Soil–Plant-Atmosphere Laboratory, with expected standard deviation < 0.3%. Paleosol pH was determined by electrode in a 1:2 mixture of ground paleosol sample to deionized water. No pre-acidification of paleosols were performed here^38^, so it is possible that paleosols with pH > 6.5 contained some amount of inorganic carbon (e.g., carbonate). All samples were analyzed in duplicate. TOC and pH values are provided in Table S4.

### Visible/near infrared spectroscopy

Visible-near infrared (VNIR) spectroscopy was used to determine the alteration mineralogy of select samples. Lithified hand samples of paleosols (approximately 200 g) were selected for analysis. An ASD FieldSpec Pro3 reflectance spectrometer in the Planetary Surfaces Laboratory at Purdue University was used to examine the reflectance spectra of samples from 0.35 to 2.50 µm. Samples were not ground or sieved before analysis. Spectra from laboratory standards of kaolinite, hematite, goethite, montmorillonite and illite from the Western Washington University Vis–NIR Spectroscopy Database were compared with spectra from hand samples to constrain the mineralogy of unknown samples. Raw spectra are provided in Table S6.

### Micromorphology

Petrographic thin sections of paleosol samples were used to classify paleosol micromorphology, estimate grain size distribution and constrain mineral composition^39^. Thin sections of oriented paleosol samples were point counted using a Swift automated stage and Hacker counting box fitted to a Leitz Orthoplan Pol research microscope. Determination of average grain size and qualitative mineralogy with error of 2% for common components^39^. A total of 1000 points on each thin section were counted (500 points for relative proportion of minerals and 500 points for determination of sand, silt and clay size fractions), thus allowing for estimates of error of 4 vol. %^40^ (Table S7). Thin section descriptions followed methodology used in Stoops (2003)^5^ (and references therein). Focus was given to pedogenic features indicative of soil forming processes (e.g., clay coatings and nodules) as well as to b-fabrics.

## Results and discussion

### Morphology and micromorphology

#### Mshap (White) profile

**Figure 4:**
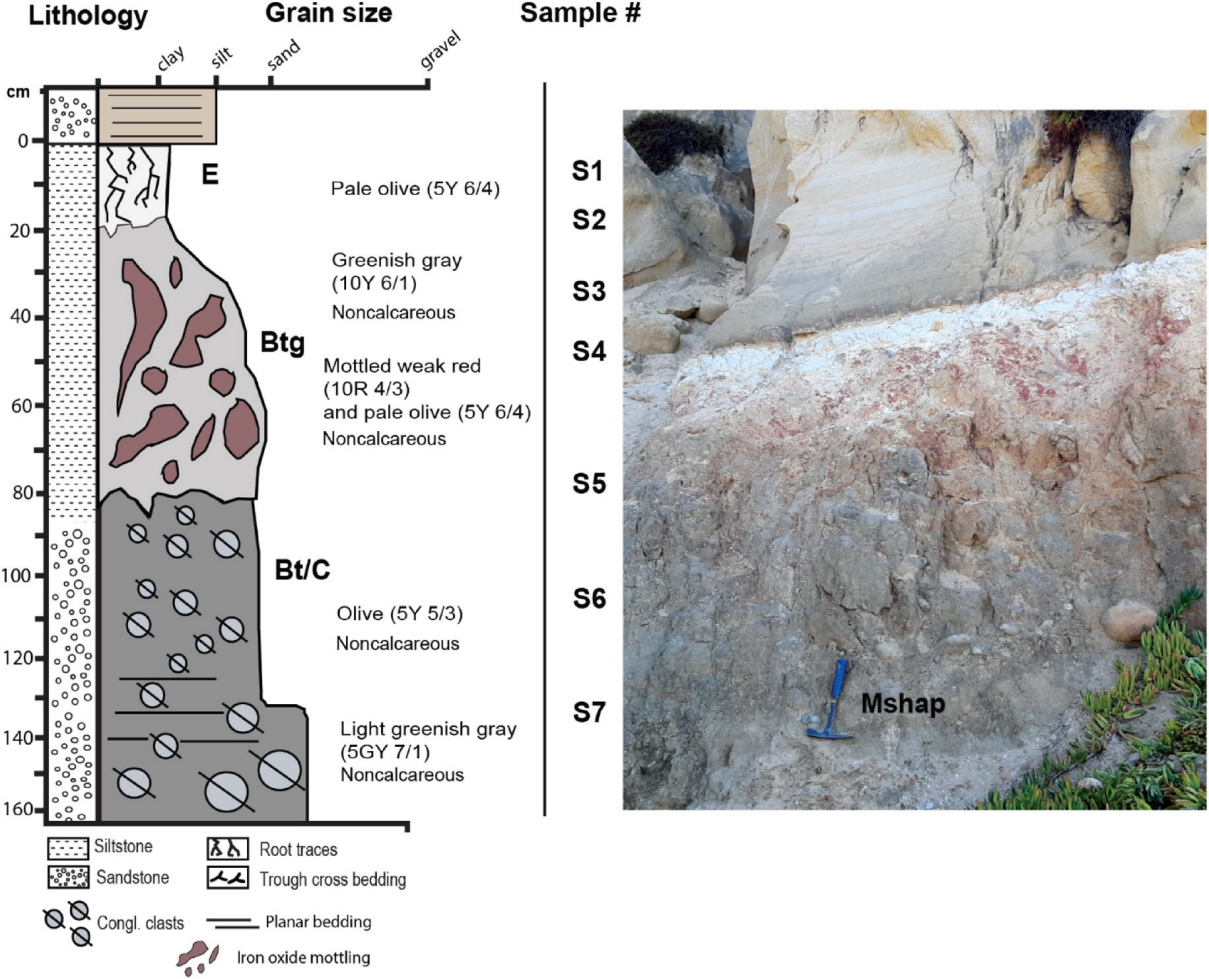
*Mshap* Ultisol paleosol at Black’s Beach, La Jolla, CA, with a kaolinitic E-horizon and a mottled Btg horizon with large drab-haloed root traces. The C horizon has rounded chert and quartzite clasts up to 15 cm in diameter that are imbricated to the west. All clasts are well rounded.

The kaolinite-rich profile analyzed in this study, herein referred to as *Mshap* ("White" in the Kumeyaay language^41^) exhibits characteristics consistent with a poorly-drained Ultisol paleosol, known as an Aquult soil in US Soil Taxonomy System^42^. This profile has a kaolinitic E-horizon that gradually transitions into a well-developed, mottled B horizon distinguished by large (10 cm length) drab-haloed root traces. The bleached E horizon subtly grades into the mottled red hues of the B horizon (Fig. 4), a transition suggesting intermittent saturation potentially caused by seasonal flooding. The deepest horizon (C) hosts a parent material of well-rounded chert and quartzite clasts, imbricated to the west, with diameters reaching up to 15 cm. The A-horizon of the original profile was likely removed by erosion during the deposition of the overlying sandstone. It appears that weathered conglomerate clasts extend deeper into the C-horizon of the profile (possibly 3–4 m), but views of such material was obscured by colluvium at the time profiles were observed (e.g., unweathered R-horizon of conglomerate at bottom of profile was covered by overburden and not visible), so it is possible that profiles are indeed ~ 3–4 m or more in vertical thickness as noted by Abbott (1981)^12^.

**Figure 5:**
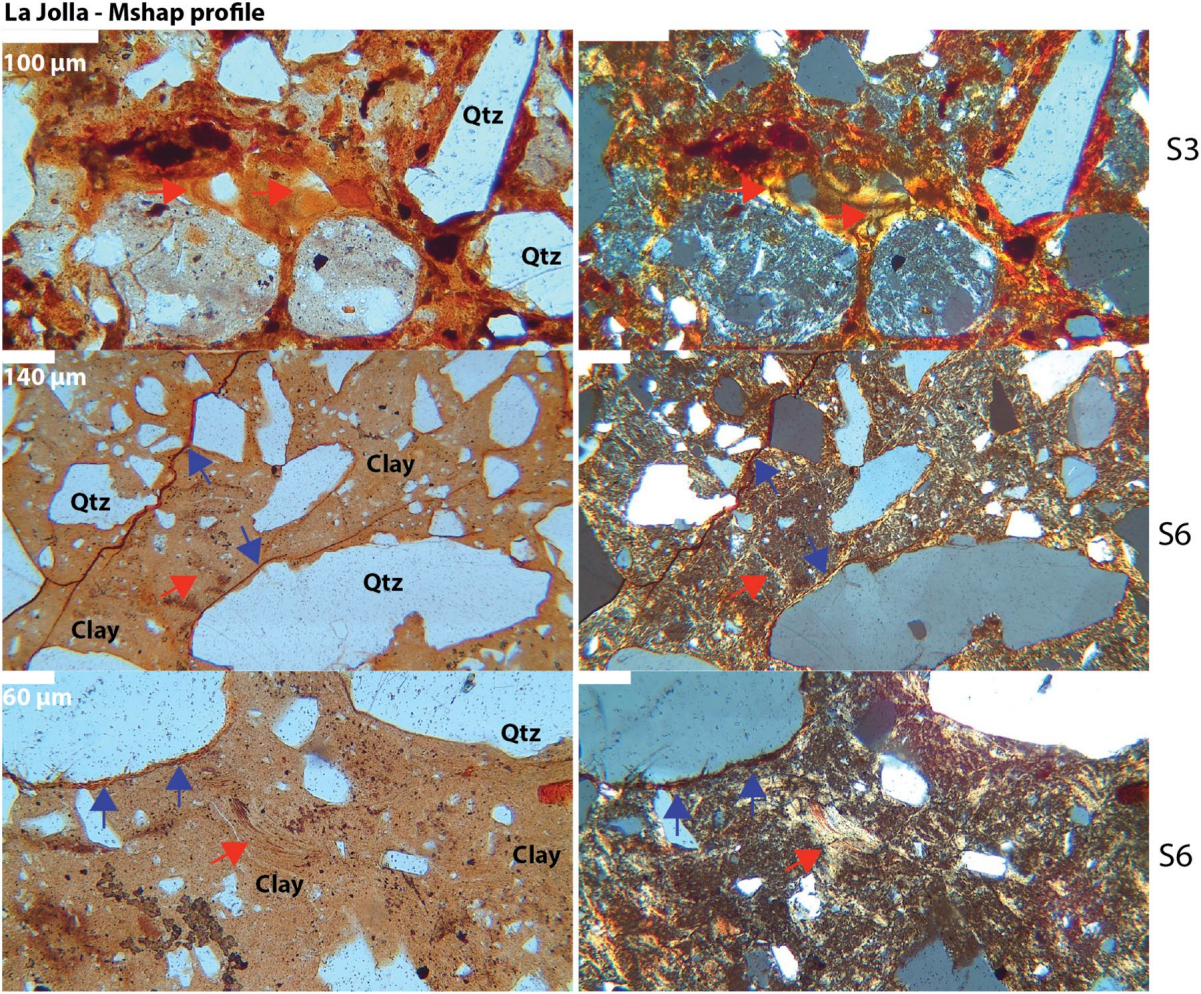
Micromorphological features of the early Eocene (55 Ma) La Jolla (*Mshap*) profile seen in plane polarized light (PPL, left column) and cross polarized light (XPL, right column). Top row is E horizon showing limpid and oriented clay coatings (red arrows) and Fe nodules (dark spots in PPL/XPL); middle row is Btg horizon showing circular striated b-fabric (red arrow) and granostriated b-fabrics with limpid and oriented clay coatings (blue arrows); bottom row is Btg horizon showing clay coatings (red arrow) and Fe hypocoatings (blue arrows). Sample nomenclature in right column can be traced across all analyses performed on samples (see Tables S1–S9).

Micromorphological observations also support the hypothesis of an Aquult-like paleosol (Table S8). The surface horizon visible in the outcrop has been identified as an E horizon, characterized by clay and Fe depletion. A few clay coating remnants are observed in several planar voids (Fig. 5), while the subsurface horizons exhibited an abundance of clay coatings and redoximorphic features (Fig. 5).

The subsurface horizons have been classified as a sequence of poorly-drained argillic (Btg) horizons, primarily due to the frequent presence of clay coatings, Fe nodules, and depletion/impregnation features (Fig. 5). The clay coatings are limpid, displaying low interference colors. These coatings are commonly found associated with planar voids and showed clear extinction lines, although signs of local disturbance such as fragmentation and poor orientation are evident. We hypothesize that pedoturbation—via the incorporation of clay coatings into a clayey groundmass—and post-burial deformation may have been contributing factors to the disturbance of clay coatings. The presence of striated b-fabrics (e.g., grano, cross, and circular) in the B horizons supports the suggestion of substantial pedoturbation processes^43^ (Fig. 5).

There was no evidence of lithological discontinuity, suggesting a continuous profile. The uniformity of the parent material across all samples is indicated by the similar c/f distribution, mineral composition, roundness, and sorting (Table S8). Quartz, which dominates the coarse fraction, displays a predominantly wavy extinction, hinting at a metamorphic origin. The intense weathering present was confirmed by the detection of fractured quartz grains infilled with kaolinite and/or Fe oxides (e.g., “runiquartz”^44^)(Table S8).

#### Hwatt (red) profile

**Figure 6:**
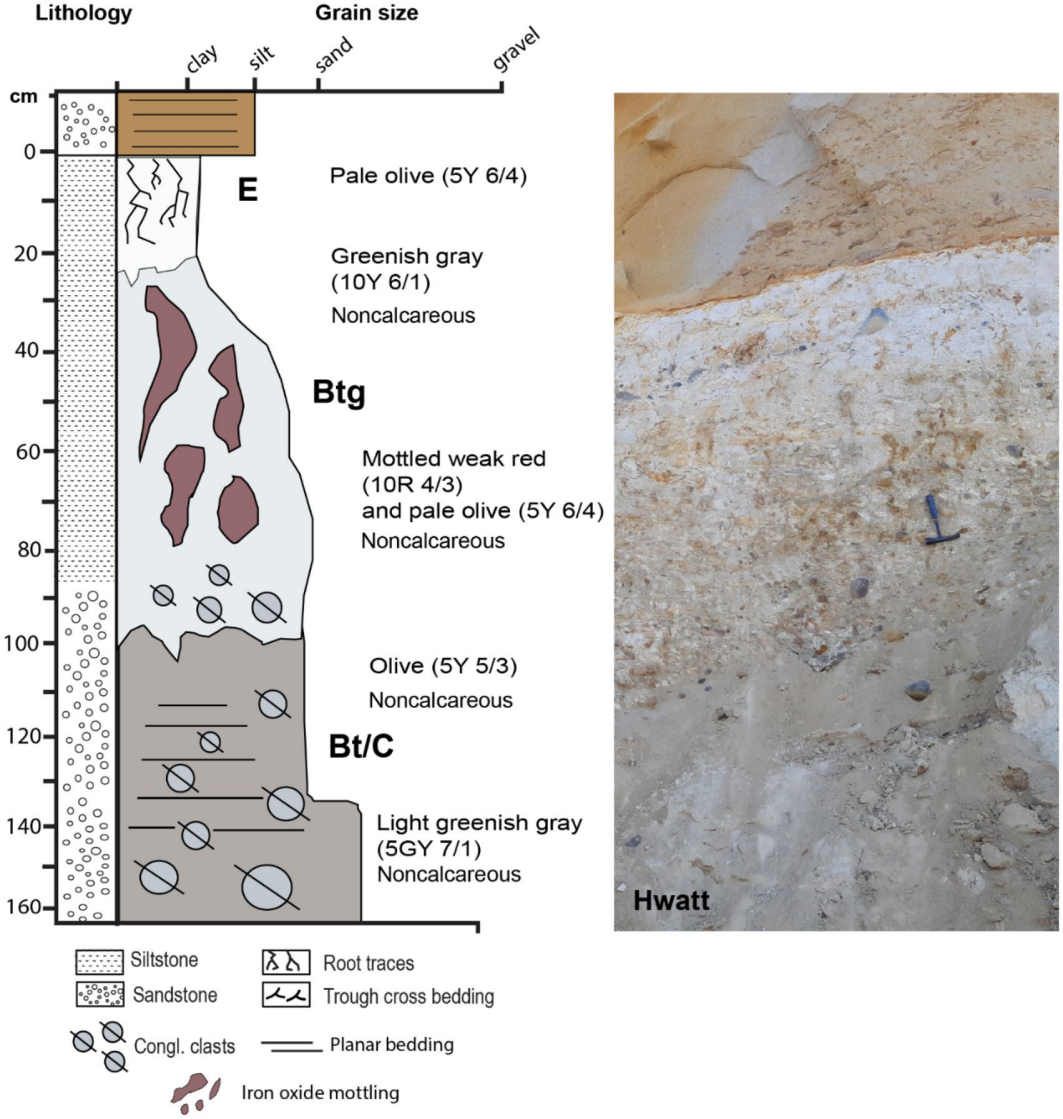
Poorly drained *Hwatt* paleosol (Aquult) at Black’s Beach, La Jolla, CA with root traces up to 2 cm in diameter and reaching to 18 cm in depth. This profile also has a kaolinitic E-horizon overlying a mottled Bw horizon with rounded qua–tzite clasts up to 15 cm in diameter.

This profile at La Jolla also resembled a poorly-drained Ultisol paleosol (Aquult) in US soil taxonomy) (Fig. 6). Because of the common and large red mottles, it is herein *referred to as Hwatt* (“Red” in the Kumeyaay language). The bleached-white kaolinitic A-horizon contained root traces up to 2 cm in diameter and reaching 18 cm in depth. This profile also has a kaolinitic E-horizon overlying a mottled Bw horizon with rounded quartzite clasts up to 15 cm in diameter and a mixture of sand and clay. The gray to white subsurface (B) horizon was consistent with poorly drained conditions indicated by the bleached surface grading into a mottled red subsurface indicative of seasonal waterlogging^45^. The C horizon contains well- rounded chert and quartzite clasts also imbricated to the west and up to 20 cm in diameter. The pair of paleosols described at Black’s Beach represent a paleocatena, two soils varying laterally (along strike) from the same ancient land surface, representing differences in paleotopography (e.g., hillslope vs. toeslope)^34^.

#### Psiiw (Green) and Hamulh (Surf) profiles

**Figure 7:**
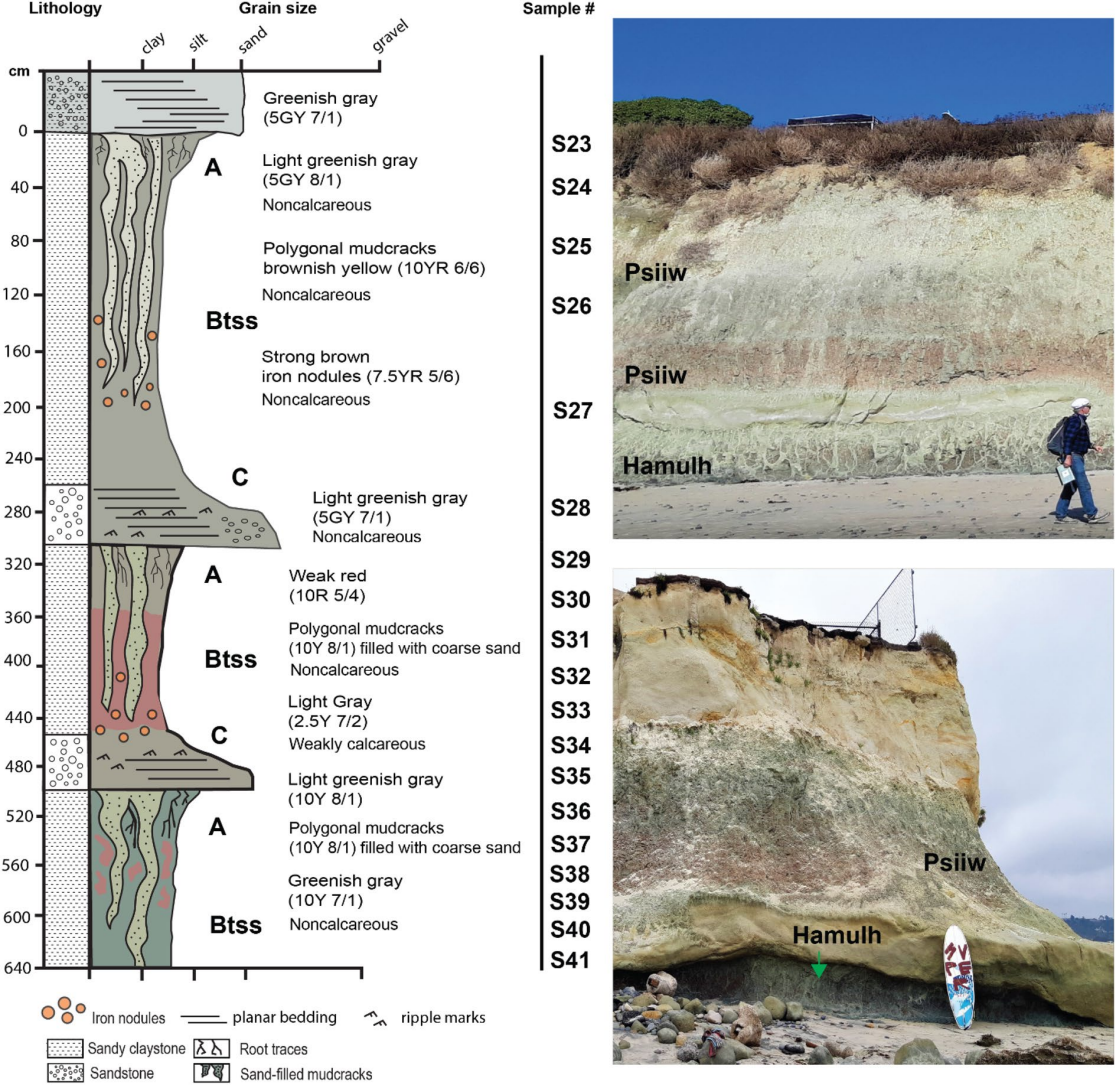
Sequence of red clay Vertisol (shrink-swell) paleosols in beach cliffs and shore platform at San Elijo Beach, Cardiff, CA. Deep (< 75 cm) coarse-sand-filled polygonal mudcracks are green/gray in color (10Y 8/1) and are present in weak red (10R 5/4) soil matrix with abundant slickenslides and Fe-bearing concretions.

This sequence of three clay-rich paleosols at Cardiff resembled a modern Vertisol (smectitic high shrink-swell soils^42^) which formed on a parent material of quartzofeldspathic sand. The uppermost two profiles, herein referred to as *Psiiw* or “green” in Kumeyaay language, overlie the basal *Hamulh* (“Surf”) pedotype that composes the shore platform and extends seaward. The weak red (10R 5/4) surface horizons contain common and massive, sand-filled polygonal desiccation features, common slickenslides oriented at random angles, and abundant drab halo root traces to 4 cm in diameter and up to 25 cm in depth. These graded into a weak red (10R 5/4) subsurface clay horizons (Bss or Bssg horizons) also with abundant slickenslides, clasts of coarse quartz sand, and Fe concretions up to 3 cm in diameter (Fig. 7). The ledge-forming C-horizon of the middle profile was a light greenish gray (10Y 7/1) noncalcareous coarse-grained quartzofeldspathic sandstone. This overlaid the basal profile, which was brick red (10 R 5/4) and also pierced with mottled green (10Y 8/1) sand-filled cracks and root traces to 5 cm in diameter with abundant slickensides. Large (75 cm depth and up to 10 cm in diameter), polygonal, sand-filled mudcracks are common in other Vertisol paleosols^46,47^.

The basal *Hamulh* paleosol profile in the shore platform extends seaward (Fig. 3) and creates “Cardiff Reef” (Fig. S1), a world-famous surfing area known for long, tapering and consistent wave formation, due in part to incision of the shore platform by the San Elijo river (Fig. S1) into the paleosol that has created a deep offshore channel located approximately 50 m south of the Cardiff study area^48^. Thus, the lowermost ~50-million-year-old paleosol is “Cardiff Reef”.

**Figure 8:**
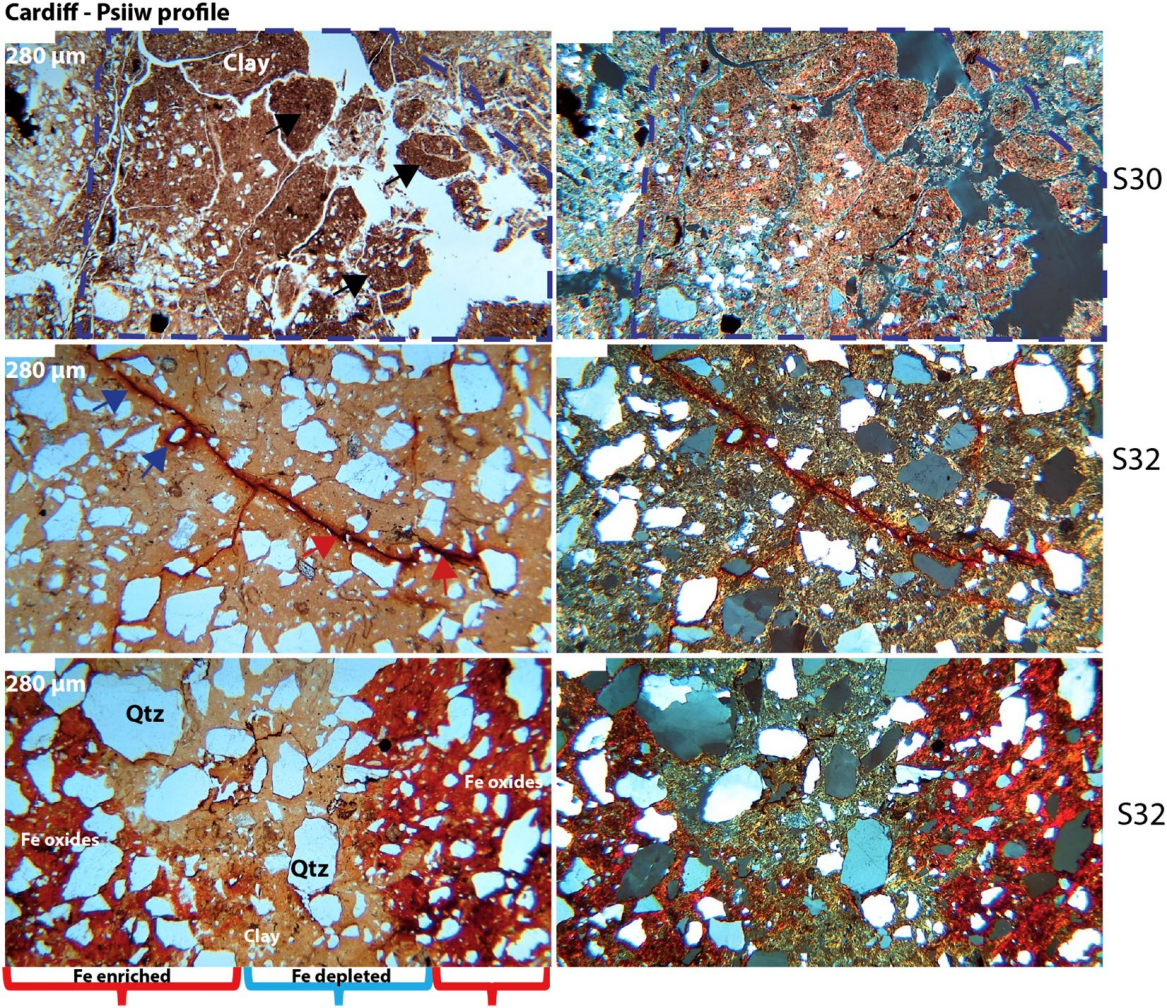
Micromorphological features of the Eocene (50 Ma) Cardiff (*Psiiw*) profile seen in plane polarized light (PPL, left column) and cross polarized light (XPL, right column). Top row shows A-horizon with subangular blocky ped structure (black arrows) and clay mineral accumulation; note well developed-pore network with finer material indicated by dashed blue line; middle row shows Btss horizon with well-developed b-fabric (yellow in PPL) and Fe-oxide lined pore network; red arrows indicate inner Fe matrix and blue arrows indicate outer diffuse Fe matrix boundary; bottom row shows Btss horizon with residual quartz and Fe enriched areas (red brackets) alternating with Fe depleted areas (blue bracket). Sample nomenclature in right column can be traced across all analyses performed on samples (Tables S1–S9).

Micromorphological observations validated interpretation of these paleosols as Vertisols (Fig. 8). Diagnostic vertic soil properties, including a large and well-developed blocky structure along with strongly striated b-fabrics, are consistently observed throughout the Bss horizons^49^ (Fig. 8).

The A horizons are characterized by a smaller blocky structure with a secondary granular structure, accompanied by a well-developed pore network resembling fine roots, which likely belonged to a grassland-type vegetation. The infilling of finer textured particles in larger pores suggested proximity to the surface Fig. 8, top row).

We identified lithological discontinuities and buried horizons, as denoted by the numerical prefix in the horizon designations and the "b" suffix, respectively. The lithological discontinuities are readily discernible due to abrupt alterations in the size, sorting, and composition of the coarse fraction. The buried horizons are identified by the sudden reappearance of A horizon properties, such as an extensively developed pore system resembling roots and material infilling.

Overall, this paleosol sequence demonstrated relatively good drainage, and only a few horizons showed redoximorphic features like Fe coatings, nodules, and an Fe-depleted groundmass (Fig. 8). Unlike the La Jolla profiles, the Cardiff profiles demonstrated a more diverse mineral composition, predominantly featuring quartz with a frequent occurrence of biotite and plagioclase. Notably, no instances of runiquartz formation are detected (Table S8).

### Visible/near infrared spectroscopy

**Figure 9:**
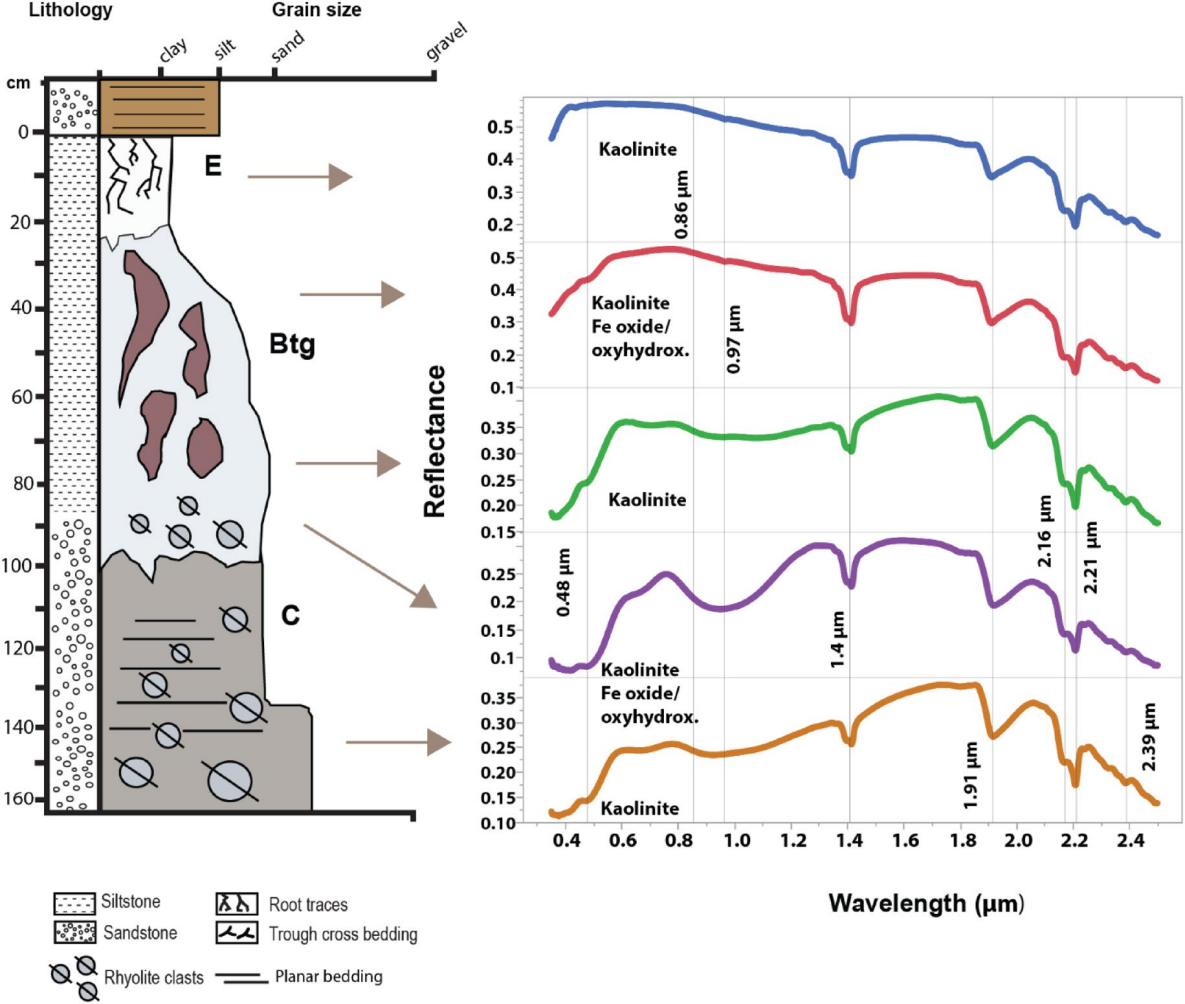
Visible-near infrared spectroscopy of the La Jolla Ultisol paleosol. Absorption features highlighted at 0.48, 0.97, 1.4, 1.95, as well as the doublet feature at 2.16 and 2.2 um, are consistent with kaolinite and Fe oxides and/or oxyhydroxides.

Analysis of the Mshap paleosol (La Jolla) showed strong absorptions with band centers near 0.5, 0.8, 0.97, 1.41, 1.9, 2.16, 2.2, and 2.39 µm (Fig. 9). We interpret these absorptions as kaolin-group minerals (kaolinite, halloysite, dickite) with contributions from Fe oxides and a Fe^3+^ -bearing phyllosilicate^50^. The absorptions at 1.41 µm are indicative of the first kaolinite overtone whereas the 1.9 µm band is from a combination tone of Al–OH bending and H–O–H stretching in H_2_O^51^ or from the presence of another hydrated phase. A shoulder exists at 2.16 as a doublet with the 2.20 µm band, which is caused by a combination tone of the OH stretch^50^ and is diagnostic of kaolinite (e.g.^52^). A band near 2.39 µm could also be consistent with OH stretching and bending combinations in a Fe^3+^ phyllosilicate, possibly due to the isomorphic substitution of Al or Fe for Si in the tetrahedral layers, or from cation bonding between tetrahedral and octahedral layers^50^.

The presence of finely crystalline Fe oxides in the lower *Mshap* profile was inferred from absorption features centered near 0.5 µm and a broad feature near 0.86 µm^53^. The C horizon had the most pronounced Fe oxide features with the largest band depth at 0.85 µm noted across all samples. Fe oxides features are mainly observed in the subsurface horizons and are absent in the surface (E horizon) samples. This suggests that the surface horizon may have been poorly drained and chemically reducing whereas the subsurface may have been well-drained and more oxidized.

**Figure 10:**
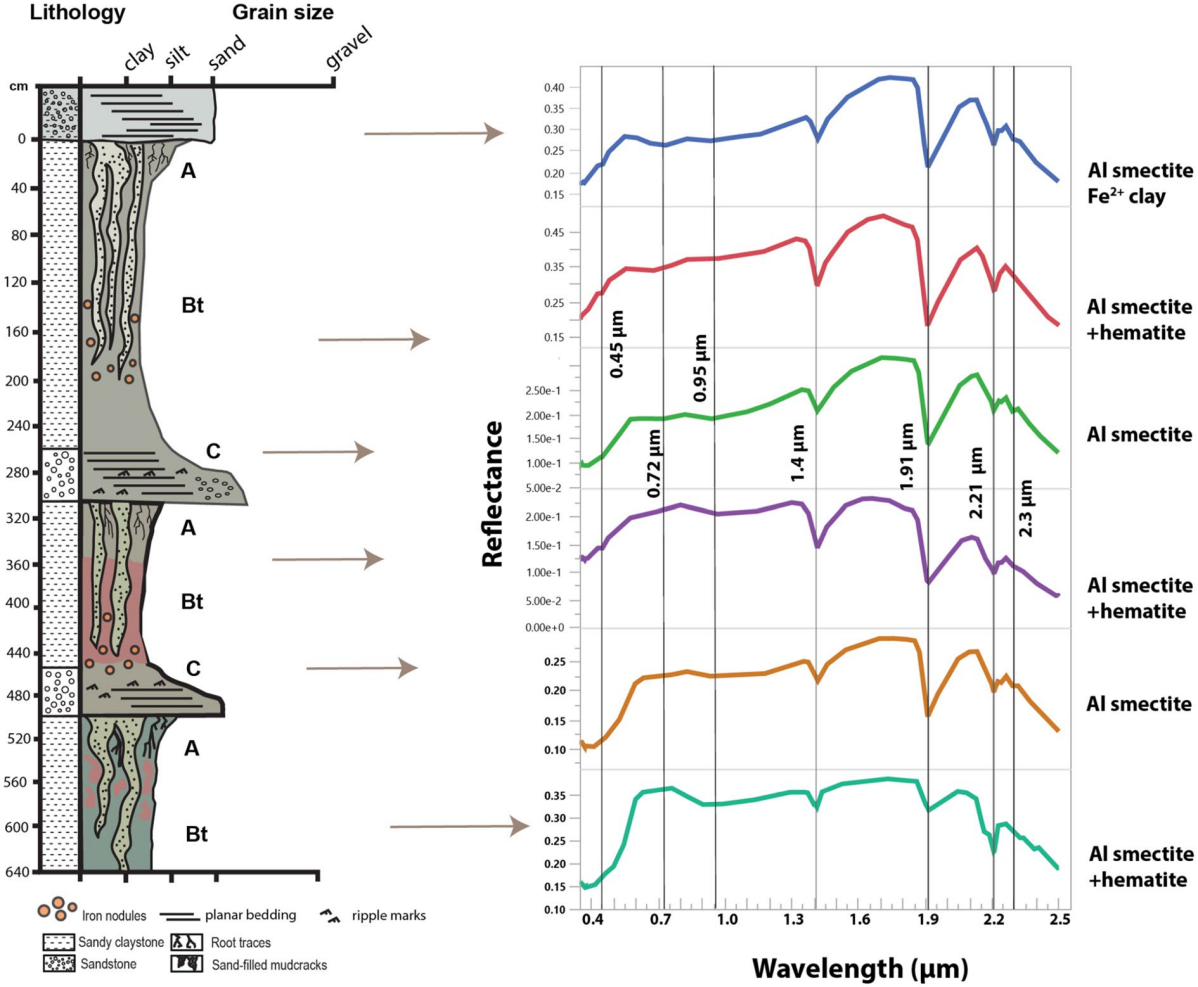
Visible-near infrared spectroscopy of three Vertisol paleosol profiles (red Hamulh and green Psiiw pedotypes) from Cardiff-by-the-sea, CA. Absorption features highlighted at 0.86, 1.4, 1.91, 2.21 and 2.3 µm are consistent with Al smectite, minor Fe smectite and hematite.

The Hamulh and Psiiw paleosols (Cardiff) had absorptions with band centers at 1.4, 1.91, 2.21 and ~ 2.35 µm (Fig. 10). The absorption features at 1.91 and 2.21 µm are consistent with a strongly crystalline Al smectite (e.g., Al montmorillonite). The absorptions at 1.4 µm and 1.9 µm are similar to kaolinite, but the kaolinite-diagnostic doublet feature at 2.16 and 2.2 µm was absent in all but one of the Cardiff samples. Instead, an absorption feature near ~ 2.35 µm is consistent with Fe^2+^ -rich phyllosilicates such as zinnwaldite and/or chamosite, or a mixed layer illite-smectite^50^. Alternatively, features closer to 2.30 µm could have resulted from a Fe^3+^ smectite such as nontronite^50^.

Despite the extensive green–red mottling in the Cardiff paleosols, Fe oxide signatures are largely absent in visible wavelengths. Only one sample, the A-horizon of the lowermost profile, had band centers near 0.5 and 0.86 µm, characteristic of Fe oxides such as hematite^53,54^. Interestingly, the lowermost profile was the reddest of the three profiles and suggested it may have been less affected by early diagenetic burial gleization^55^. This process may have converted a significant portion of the Fe oxides and oxyhydroxides from the ferric state to a drab-colored ferrous state, and since ferrous iron is more soluble, may have resulted in depletion of total iron in the profiles^55^. This may be why we did not see strong Fe oxide signatures in most samples despite the inferred presence of ferric iron characteristic of deeply weathered soils^56^.

### Chemical weathering trends

**Figure 11:**
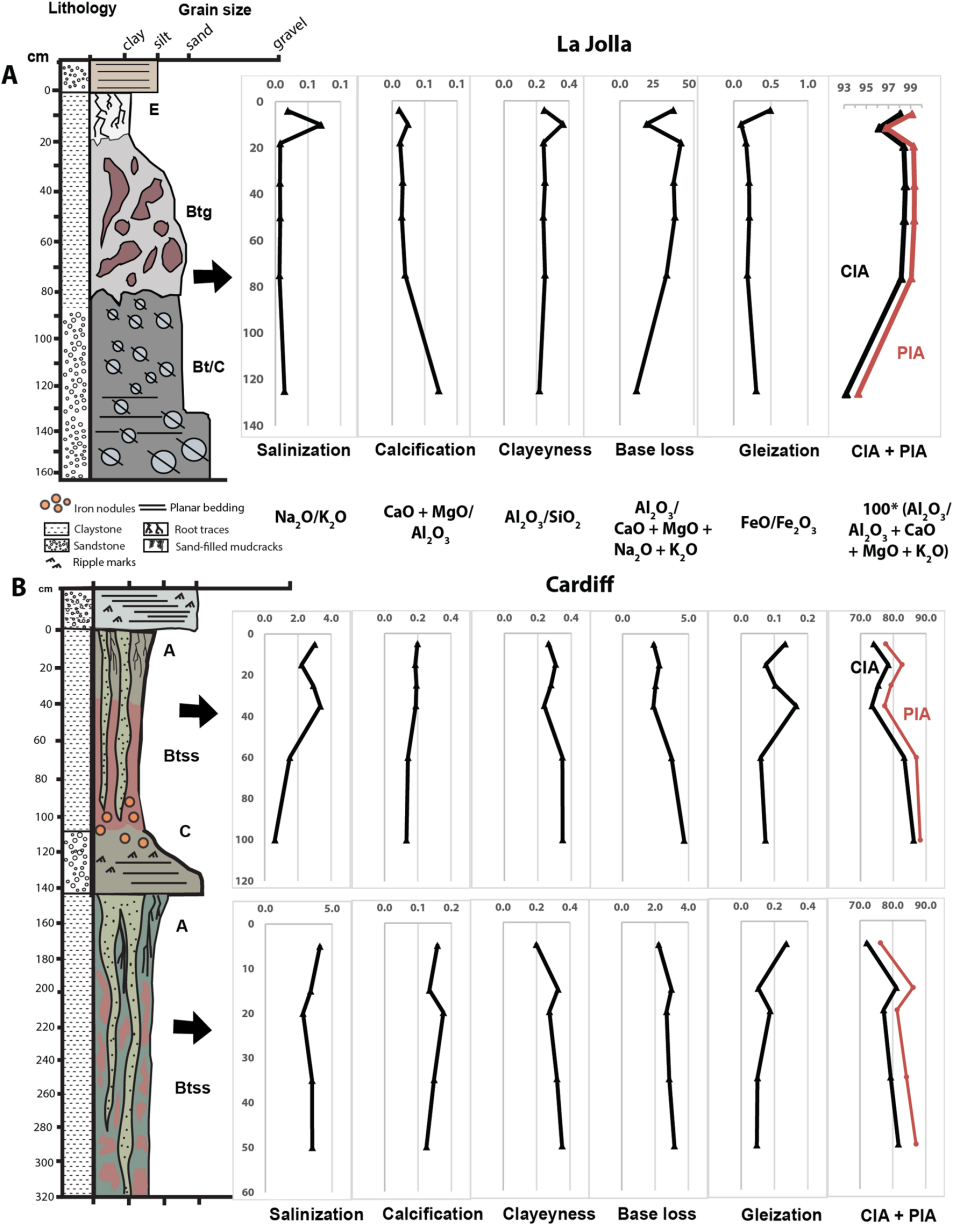
Geochemical trends of paleosols from La Jolla and Cardiff-by-the-Sea, CA. (**A**) Geochemical trends with depth in a kaolinitic Ultisol from Black’s Beach, La Jolla, CA; and (**B)** Vertisol (high shrink-swell) paleosols from San Elijo Beach, Cardiff by-the-sea, CA. CIA, Chemical index of alteration (100*[Al_2_O_3_/Al_2_O_3_ + MgO + CaO + K_2_O]); PIA, Plagioclase index of alteration (100*[Al_2_O_3_-K_2_O/Al_2_O_3_ + MgO + CaO-K_2_O]).

Paleosols at both localities showed chemical weathering trends consistent with extensive leaching and subaerial alteration (Fig. 11). The *Hwatt* Ultisol-like paleosol (La Jolla) showed only slight salinization (Na_2_O/K_2_O) and calcification (CaO + MgO/Al_2_O_3_) with values less than 0.15 (Fig. 11A). On the other hand, we observed moderate clayeyness (Al_2_O_3_/SiO_2_) in the uppermost horizon with values up to 0.4 that decreased to 0.2 in the subsurface (Bt and C) horizons. Base loss followed a similar trend where the highest values (~ 40) are noted in the near-surface horizons and decreased to values less than 20 in the C horizon. Gleization, indicative of waterlogging before burial, was greatest in the surface (E) horizon and decreased with depth. Low salinization and calcification values (~ 0.1) are noted and are common in Ultisols of wet climates where precipitation exceeds evapotranspiration^34^. Clayeyness and base loss are highest in the near-surface horizons of the paleosol, indicative of subaerial alteration and leaching, but overall values are less than would be expected for a more deeply weathered Oxisol. Gleization values of ~ 0.5 in the A-horizon also suggest waterlogging conditions before burial and are consistent with seasonal saturation by surface water. A decrease of FeO/Fe_2_O_3_ in the subsurface horizons suggests perched surface water rather than groundwater was responsible for the seasonal waterlogging conditions^55^.

The Cardiff Vertisol paleosols (*Psiiw and Hamulh*) had salinization and calcification values up to ~ 4 and 0.2, respectively, with the highest values in the A horizons of both profiles (Fig. 11B). Moderate salinization suggests that precipitation was not adequate to remove most Na_2_O, especially when compared to the low salinization values of the La Jolla (*Hwatt)* profile. Low calcification values (up to 0.2) are similar to the Hwatt profile, suggesting an absence of pedogenic carbonate. Vertisols of wet climates such as those examined here (MAP >  ~ 1000 mm) do not typically contain pedogenic carbonate whereas Vertisols of dry climates (MAP < 1000 mm) can accumulate pedogenic carbonate in subsurface (i.e., Bssk) horizons^57^, leading to increased calcification values^34^. On the other hand, base loss in the Cardiff Vertisols was an order of magnitude lower than the Hwatt paleosol (base loss values of 1–4 vs. 40). These base loss values are consistent with other observations of Cambrian Vertisol paleosols from South Australia^58^ and suggest lower weathering intensity compared to the *Hwatt* profile. Lastly, gleization was highest in the paleosurface horizons of both profiles, suggesting either seasonal saturation during pedogenesis or burial-induced diagenesis such as burial gleization^59^. Burial gleization is envisaged as the reduction of Fe by anaerobic microbes shortly after burial^60^. In both cases, accumulation of FeO is limited to the near-surface horizons (e.g., the paleosurface).

### Chemical index of alteration

The geochemistry of the Mshap paleosol (La Jolla) showed extensive depletion of mobile cations (Ca, Mg, K, Na) and a chemical index of alteration minus potassium (CIA-K) of > 98 in the near-surface horizon (Fig. 11A). The profile was nearly devoid of all mobile cations and was significantly enriched in Si and Al. The paleosurface horizons (A and Bt) had the highest CIA-K observed in the study with average values of ~ 99 that decreased to ~ 93 in the subsurface (Bt/C) horizon. These high CIA values are indicative of nearly complete kaolinitization, typical of highly weathered soils and paleosols^13,61^.

The Hamulh and Psiiw Cardiff paleosols sequence (50 Ma) was less intensely weathered relative to the Eocene La Jolla paleosol (55 Ma), though with significant depletion of Ca, Mg, K, and Na, and CIA-K values ranging from ~ 77–88 (Fig. 11B). The CIA was greatest in the lower A and upper Bss horizons of both profiles. Though not as intensely weathered relative to the La Jolla paleosol, the accumulation of Fe oxides and massive vertic features including sand-filled cracks also indicate extensive leaching under a warm, humid and seasonally dry climate. The less intense weathering of Cardiff paleosols is supported by micromorphological observations where biotite and plagioclase are detected, while only quartz was detected in the La Jolla paleosols.

It should be noted that CIA can be influenced by parent lithology, such that felsic rocks often have higher starting values of CIA in rocks that are not altered (e.g. values of 45–50) compared to starting values for mafic rocks (35–40)^13,62^, so interpretations of CIA should take into consideration the composition of the protolith. It is challenging to tease apart these differences in the profiles, however, because the parent materials are predominantly felsic but also have a mafic component (e.g., coarse quartzofeldspathic sand protolith of the Cardiff paleosols and an exotic conglomerate protolith for La Jolla paleosols).

When comparing trends in CIA in the profiles at Cardiff and La Jolla, the Cardiff CIA values are both lower and more variable throughout the sequence when compared to the higher and more uniform CIA values in the La Jolla profiles. These differences are likely due to differences in the intensity/duration of weathering (La Jolla Ultisols are more weathered than Cardiff Vertisols and thus have more uniform CIA) and rate of sedimentation (Cardiff paleosols are in a sequence, suggesting multiple episodes of sedimentation and weathering, whereas La Jolla profiles are present as individual profiles).

La Jolla Ultisol paleosols had total organic carbon (TOC) ranging from 0.026 to 0.079 (± 0.003) wt. % and pH ranging from 3.523 to 6.283 (± 0.018). (Table S2). Like modern soil profiles, the organic carbon content was enriched in the surface horizons of paleosols (E horizon) and subsequently depleted in the lower horizon (C horizon). It should be noted that paleosol pH is often compromised by late-stage groundwater alteration, which can reset the original pH^37^, so caution is needed for primary interpretation of paleo-pH reconstruction from direct measurements of pH. However, modern Oxisols and Ultisols are characterized by low pH as a result of intense weathering and the generation of organic acids^44,63,64^, so perhaps the pH values we measured represent minimal post-diagenetic groundwater alteration and thus reflect the paleo-pH of the La Jolla profile. Alternatively, there could have been late diagenetic groundwater alteration with acidic fluids, but we find this hypothesis less likely due to the dearth of evidence representing early diagenetic intense weathering conditions.

Diagenetic additions of recent/modern organic C can inflate the so-called “preserved” organic C^36^, but enrichments of TOC in uppermost horizons of paleosols are consistent with preservation of endogenous organic C^60^. Thus, it is possible that organic C is preserved in the La Jolla profile, though additions of small amounts of geologically recent/ modern carbon are possible and perhaps likely.

Cardiff Hamulh and Psiiw Vertisol paleosols had TOC ranging from 0.019 to 0.074 (± 0.003) wt. % and pH ranging from 7.373 to 8.907 (± 0.023) (Table S2). Like modern soil profiles, again the organic carbon content was enriched in the surface horizons of paleosols (A and Bt) and subsequently depleted in the lower horizons (C horizon). The Cardiff pH results (pH > 8 in some Btss/C-horizons, Table S4) suggest possible late-stage groundwater alteration (e.g., saltwater brines in shore platform) to increase alkalinity in these profiles, as it is unlikely that Vertisols had such alkaline pH during soil formation unless they formed in relatively dry climates (MAP <  ~ 1000 mm) which would allow for the formation pedogenic carbonate. Since there was no pedogenic carbonate observed in any of the Cardiff profiles, it is likely that the elevated pH is due to late diagenesis^37^. The diagenetic history of these paleosols is outlined in the following section.

### Diagenetic alteration

Burial diagenesis is commonly observed in paleosols and particularly affects pre-Quaternary paleosols. The main diagenetic processes can range from minor (burial decomposition of organic matter) to severe (contact metamorphism)^45^. Four types of diagenetic alteration that have affected paleosols in this work are burial reddening, illitization of smectite, burial gleization, and burial decomposition of organic matter.

The diagenetic process of burial reddening refers to the dehydration of Fe oxyhydroxides (e.g., goethite, ferrihydrite) and subsequent formation of Fe oxides such as hematite^5^. This most likely affected the Cardiff Vertisol profiles (Fig. 3). Modern smectite-rich Vertisols are commonly dark brown to orange in color due to accumulation of goethite and Mn-bearing phases^42,57^ rather than the brick-red Cardiff paleosol profiles. Alternatively, the Fe oxide minerals may not have formed from burial diagenesis and instead formed during pedogenic alteration before burial, but such accumulation of Fe oxide and subsequent red color is more characteristic of well-drained, highly weathered, non shrink-swell soils (Ultisols, Oxisols)^65^ rather than Al/Fe smectite-bearing Vertisols^44,66^.

Illitization of smectite (potash metasomatism) is common in paleosols that are subject to burial diagenesis^62,67^ and involves the incorporation of K into the crystalline structure of kaolinite or smectite clays such as montmorillonite and nontronite^68,69^. Diagenetic K enrichments can be addressed by ternary plots of Al_2_O_3_—(CaO* + Na2O)—K_2_O (e.g., A–C*N–K plot) versus CIA, where C* accounts for the presence of carbonates and/or apatite^62,70^. Possible evidence for illitization of smectite in the lowermost Cardiff profile included VNIR absorbance features at ~ 2.35 and 2.44 microns (Fig. 8), which is possibly consistent with mixtures of kaolinite and illite^50,71^ or a mixed layer illite–smectite clay. Alternatively, illite can be derived from the weathering of muscovite and not formed from metasomatic processes^71^, so caution is necessary for interpreting the origin of illite. In any case, further analytical work (e.g., quantitative x-ray diffraction) is needed to support the hypothesis of diagenetic illite in profiles examined in this work.

The striking green–red mottling observed in the paleosurface horizons of the Cardiff paleosols likely resulted from alteration after burial. Burial gleization, a form of early diagenesis, is thought to result from microbial reduction of Fe oxides under hypoxic or anoxic conditions shortly after burial^59^. It most commonly manifests as green-gray color mottling and is restricted to the paleosurface horizons where organic matter is concentrated (e.g., A-horizons). It can be distinguished from groundwater alteration or other primary redoximorphic features by its confinement to the A-horizon of paleosols^59^, whereas groundwater alteration from a fluctuating water table introduces gley colors to the lower parts of the profile (B and C horizons)^34,55^.

Burial decomposition of organic matter affects most all paleosols, but is more pronounced in those forming under oxidizing, well-drained conditions before burial^35^. This phenomenon, which is thought to be a form of early diagenesis, can lead to severe losses of organic carbon in profiles that were once rich in organic matter. We observed evidence of burial decomposition of carbon because the TOC in all samples (< 0.1 wt.%) was two to three orders of magnitude lower than would be expected in comparable modern Ultisols and Vertisols of subtropical climates^35,72^ . Redox state before burial, inferred from the ratio of FeO/ Fe_2_O_3_, is related to the TOC content of paleosols^35^. Generally, paleosols forming under reducing conditions (FeO/ Fe_2_O_3_ > 0.5) have significantly higher TOC relative to more oxidized profiles with FeO/ Fe_2_O_3_ < 0.5^35^. Samples with higher FeO/Fe_2_O_3_ such as the surface (A) horizon of the La Jolla paleosol (*Hwatt*, Fig. 11) had significantly more organic carbon (~ 0.07 wt. TOC %) (Table S4) relative to samples with lower FeO/ Fe_2_O_3_, (~ 0.03 wt. %) providing additional evidence that redox state before burial is related to organic preservation in paleosols.

A summary of the soil forming factors is provided in Table 1. Kaolinite-bearing *Hwatt* and *Mshap* profiles at La Jolla are similar to Aquults in US Soil taxonomy, with bleached surfaced horizons and weakly developed (Bw) subsurface clay horizons characteristic of a seasonally wet coastal lowland landscapes. Similar soils with CIA > 95 and bleached surface horizons form under warm, humid and everwet conditions characteristic of single-tier tropical forests. Poorly drained *Hwatt* paleosols could have formed beneath a seasonally dry swamp forest in a wet coastal lowland whereas the *Mshap* profiles on well-drained alluvial terraces supporting a single tier tropical forest. *Psiiw* and *Hamulh* Vertisol paleosols at Cardiff likely formed under warm, humid and seasonally dry conditions on a parent material of quartzofeldpathic silt/ sand and possibly supported a tropical seasonally dry woodland.

**Table 1:** Summary of La Jolla and Cardiff paleosol interpretations.

Pedotype	Location	Soil Taxonomy	FAO Map	Australia	Climate	Organisms	Topography	Parent material
“Hwatt” “Red”	La Jolla	Aquult	Dystric Gleyisol	Humic Gley	Not diagnostic	Seasonally dry swamp forest	Seasonally wet coastal lowland	Conglomerate
“Mshap” ‘White”	La Jolla	Aquult	Dystric Cambisol	Brown Earth	Humid, everwet	Tropical forest, single tier	Well-drained alluvial terrace	Conglomerate
“Psiiw” “Green”	Cardiff	Vertisol	Vertisol	Red Clay	Warm, humid, seasonally dry	Seasonally dry tropical woodland	Well-drained coastal terrace	Quartzofelspathic silt and sand
“Hamulh” “Surf”	Cardiff	Vertisol	Vertisol	Red Clay	Warm, humid, seasonally dry	Seasonally dry tropical woodland	Well-drained coastal terrace	Quartzofelspathic silt and sand

### Geochemical climofunctions and implications for early Eocene climate

Paleoclimate estimates relating CIA-K (chemical index of alteration minus potassium) to mean annual precipitation^19^ are shown in Table 2. Also shown are “RF-MAP 1.0”^22^ paleoprecipitation estimates based on paleosol geochemistry using a predictive random forest algorithm that accounts for true prediction error (root mean square error of prediction, RMSEP)^22^. We also constrained paleotemperature using the salinization index (K_2_O + Na_2_O/Al_2_O_3_) outlined in Sheldon et al. (2002)^19^. Samples from the Bt horizon of the Paleocene-Eocene Thermal Maximum (PETM, 55 Ma) Mshap profile in La Jolla yielded mean annual temperature (MAT) estimates of 17.5–17.7 °C  ± 4.4 °C and mean annual precipitation (MAP) of 1487–1558 mm ± 299 mm, consistent with a humid subtropical climate. The early Eocene Climatic Optimum (EECO, 50 Ma) Psiiw and Hamulh profiles in Cardiff yielded MAT estimates of 19.8–20.6 °C ± 4.4 °C and mean annual precipitation (MAP) of 1227–1014 ± 299 mm/yr, also consistent with a subtropical humid climate. Evidence for seasonality of precipitation was inferred from vertic features including large sand-filled mudcracks, suggesting a summer-dry EECO climate.

**Table 2:** Geochemical climofunctions from A and B horizons of early Eocene (55 Ma) paleosol from La Jolla, CA and Eocene (50 Ma) paleosols from Cardiff, CA.

Location	Age (Ma)	Depth (cm)	Horizon	CIA-K	CALMAG	Paleotemp. (C° /yr)	Error	Paleoprecip. (mm/yr)	Error	CALMAG paleoprecip. (mm/yr)	RF-MAP 1.0 (mm/yr)	Error
La Jolla	55	4	Bt	99	–	17.5	4.4	1558	299	–	1255	395
La Jolla	55	10	Bt	97	–	17.7	4.4	1487	299	–	1390	395
La Jolla	55	18	Bt	99	–	17.5	4.4	1562	299	–	1332	395
Cardiff	50	25	Btss	79	84	20.9	4.4	1054	299	1467	658	395
Cardiff	50	35	Btss	77	84	20.8	4.4	1014	299	1473	739	395
Cardiff	50	60	Btss	87	88	19.9	4.4	1227	299	1552	1210	395
Cardiff	50	15	Btss	86	88	19.8	4.4	1207	299	1565	1275	395
Cardiff	50	20	Btss	81	85	20.6	4.4	1096	299	1494	732	395
Cardiff	50	35	Btss	84	87	20.0	4.4	1160	299	1541	1086	395

Chemical index of alteration minus potash (CIA-K) (Sheldon et al., 2002) and RF-MAP 1.0^19^ were used to calculate estimates of paleoprecipitation during soil formation. Transfer functions outlined in Sheldon et al. (2002)^19^ are based on a database of modern soils (R^2^ = 0.72, prediction error = 299 mm (prediction error from Lukens et al., 2019)^22^; RF-MAP1.0, also based on a database of modern soils, has prediction error of 395 mm^22^. The CALMAG weathering index, designed for use with Vertisol paleosols^21^, is defined as Al_2_O_3_/(Al_2_O_3_ + CaO + MgO) × 100 and the resulting transfer function (R^2^ = 0.9, s.e. = 108 mm) was compared with CIA-K paleoprecipitation estimates. Paleotemperature estimates are from Sheldon et al. (2002)^19^ using the salinization index (K_2_O + Na_2_O/Al_2_O_3_) (R^2^ = 0.37, s.e. = 4.4 °C). S.e, standard error.

It should be noted that the paleotemperature proxy based on salinization index (K_2_O + Na_2_O)/Al_2_O_3_) shows a less robust relationship (R^2^ < 0.37) compared to the CIA-K MAP proxy^19^ and indicates high root mean standard error and thus likely high root mean standard prediction error (RMSPE). Furthermore, it may potentially underestimate paleotemperature because extensive rainfall often associated with higher MAT removes Na and K^19,22^.

Paleoprecipitation in the Cardiff Vertisols was estimated using the RF-MAP 1.0 proxy^22^ and the CALMAG transfer function, specifically designed for use in Vertisol paleosols^21^. As noted above, CIA-K MAP estimates ranged from 1227 to 1014 ± 299 mm/year and are comparable to RF-MAP 1.0 values of 1275–732 ± 395 mm/year. The Cardiff Vertisols had higher estimated CALMAG MAP values ranging from 1494 to 1565 ± 108 mm/yr. This is consistent with the phenomenon of underestimation of paleoprecipitation using CIA-K in Vertisols of wet climates^21^. Together, these estimates suggest a possibly everwet tropical PETM paleoclimate that became warmer and drier in the EECO. Paleoclimate estimates of both localities therefore provide additional evidence of multiple episodes of warm and wet tropical Eocene climates.

It should be noted that tectonic conditions during the study interval could have contributed to patterns of moisture transport. Early Eocene segmentation of the California borderland may have occurred, with a number of submerged areas and islands apparently present^73^, which altogether provides an understanding the wider picture of offshore islands of the future Transverse Range, Mogollon Highlands inland and the Nevadaplano well to the north of the study location. Such offshore islands could have presumably caused a rain shadow; however, it does not seem more significant than the modern Channel Islands^73,74^.

**Figure 12:**
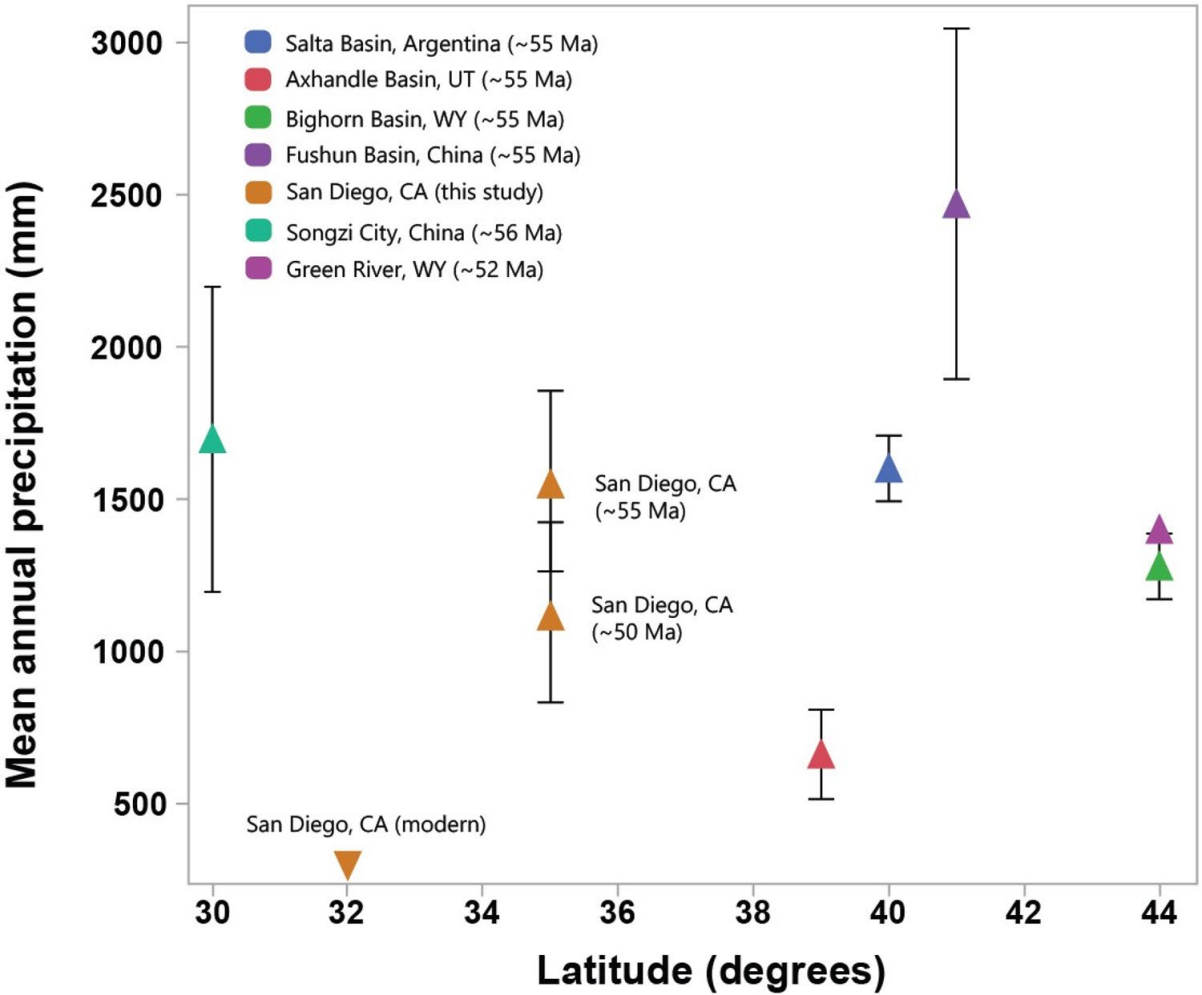
Comparisons of paleoprecipitation versus latitude during early Eocene greenhouse climates (~ 55–50 Ma) and comparisons with modern climate in present-day San Diego, CA. Mean annual precipitation (MAP) estimates are from Argentina paleosols^6^, Axhandle Basin, Utah paleosols^77^, Bighorn Basin, Wyoming paleosols^20^, fossil plants of Fushun Basin, China^78^; fossil pollen near Songzi City, China^79^, and fossil plants of the Green River Basin, Wyoming^8^. Error on paleolatitude is approximately ± 5°. Note paleolatitude of Argentina site is ~ 40° S. Average CIA-K MAP proxy values^19^ are plotted for both San Diego sites.

The range of early Eocene rainfall and temperature estimates presented in this work are consistent with previous calculations of paleotemperature and paleoprecipitation from early Eocene fossils and paleosols (Fig. 12). These include estimates of paleotemperature from PETM paleosols in Argentina (15 °C ± 4.4 °C)^6^ detrital and authigenic kaolinite from eastern California’s Sierra Nevada (23.2 °C  ± 6.4 °C)^75^ and fossil leaf-margin derived analysis from Bighorn Basin, Wyoming of 19.8 ± 3.1 °C^76^. Additional estimations from fossil flora of the middle Wasatchian (~ 52 Ma) in Wyoming range from MAT of 21 °C and MAP of nearly 1400 mm^8^ are closer to the Cardiff Vertisol paleosols (Table 2). From a mineralogical perspective, the presence of potentially abundant kaolinite in La Jolla paleosols (Fig. 8) is also similar to PETM paleosols from Texas^14^, Argentina^6^ and Australia^7^.

The seasonally dry and Al smectite- rich Cardiff Vertisols are consistent with a decrease in MAP (Table 2) after the PETM and seasonality of precipitation at paleolatitudes of 35–45° N during the EECO^80^. Such seasonality of precipitation is also consistent with previous EECO observations from fluvial sediments^81,82^, paleosols^18^, and fossils^83^. Estimations of climate from early Eocene coastal paleosols of Southern California therefore provide a new locality for paleoclimate reconstructions as well as for quantifying the nature and intensity of early Eocene weathering on land in present-day southern California.

## Conclusion

Deeply weathered paleosols from the Eocene (55 Ma) Mt. Soledad Formation and the Eocene (50 Ma) Delmar Formation near San Diego, CA provide new evidence of a subtropical humid climate in southern California during and after the Paleocene-Eocene thermal maximum. Early Eocene (~ 55 Ma) kaolinitic Ultisol paleosols developed in volcaniclastic conglomerates were subject to intense subaerial alteration and leaching with CIA-K near 99, MAT of ~ 17 °C  ± 4.4 °C and MAP of ~ 1500 ± 299 mm, characteristic of severe weathering under subhumid tropical conditions for tens of thousands of years. Geologically younger Early Eocene (50 Ma) smectitic Vertisol paleosols developed atop coarse sandstones are also intensely weathered (CIA > 80) and yield MAT estimates of ~ 20 °C ± 4.4 °C but with lower estimated MAP (~ 1100 ± 299 mm) and evidence for seasonality of precipitation. This may have been due to a decline in weathering intensity over ~ 5 Ma, or a difference in soil-forming factors other than climate such as topography or time of formation. Paleosols examined in this work represent maximum sea level regression in the Eocene of present-day southern California and also reveal a CO_2_ greenhouse spike of tropical weathering conditions on land surfaces.

### Supplementary Information

Supplementary Information 1.

Supplementary Information 2.

## Data Availability

All data generated or analyzed during this study are included in this published article [and its supplementary information files].
