# Supplementary material for: Eocene (50–55 Ma) greenhouse climate recorded in nonmarine rocks of San Diego, CA, USA
Source: Sci Rep. 2024 Jan 31;14:2613. doi: 10.1038/s41598-024-53210-0 (PMC10830502; doi:10.1038/s41598-024-53210-0)
Supplement: Supplementary file 1 — Supplementary Information 1. [file 41598_2024_53210_MOESM1_ESM.docx]

Supplementary information for “**Eocene (50-55 Ma) greenhouse climate recorded in nonmarine rocks of San Diego, CA, USA”**


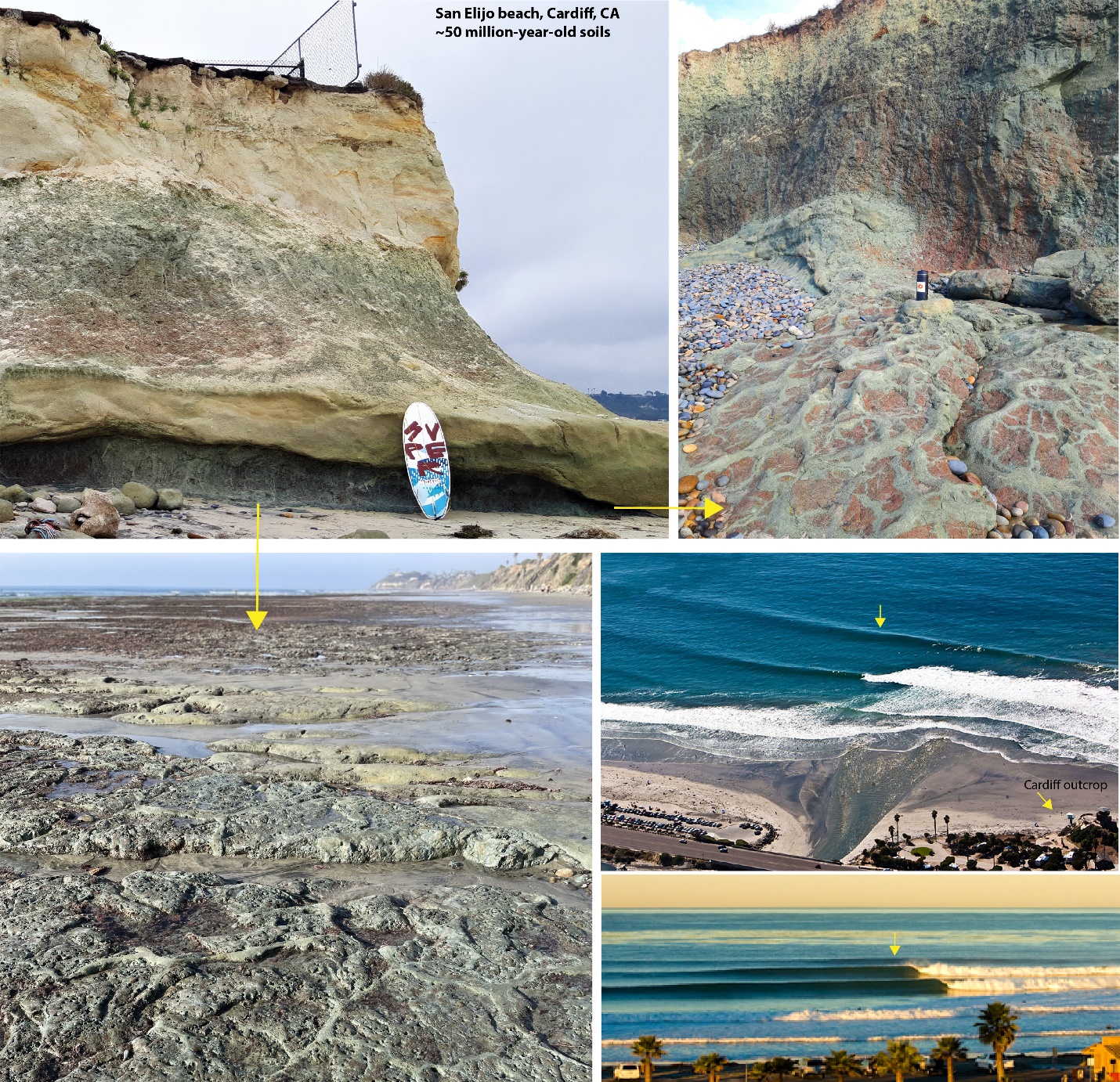


**Figure S1. Study location at San Elijo State Beach, Cardiff-by-the-Sea, CA showing a sequence of early Eocene (~50 million-year-old) deeply weathered Vertisol paleosols.** The basal profile (“Hamulh”, or “Surf” in the Kumeyaay language) with green/gray sand-filled polygonal mudcracks in the shore platform (upper right and lower left) extends to sea (yellow arrows) and creates the offshore “Cardiff Reef” (bottom right, courtesy of Tom Cozad)


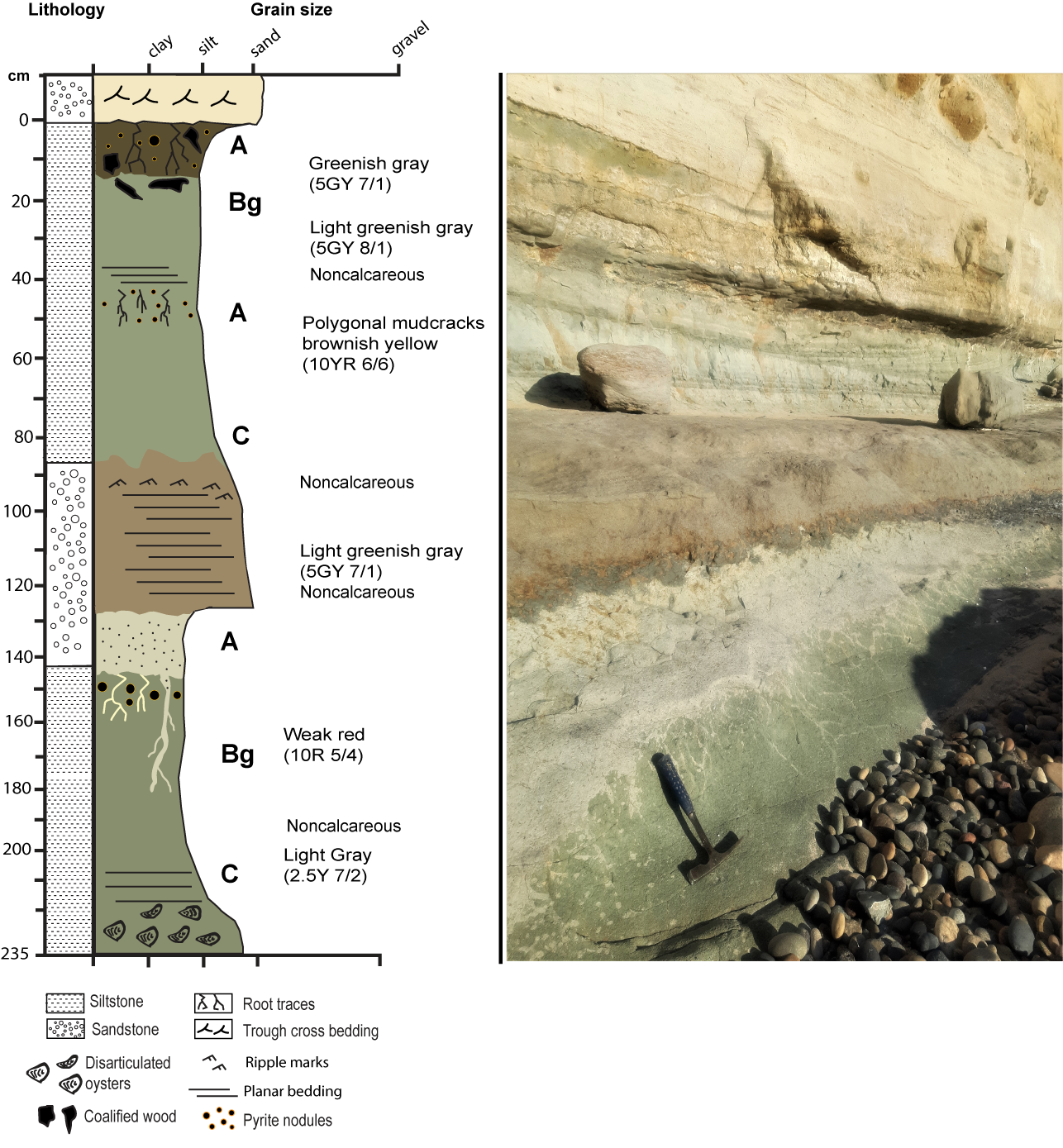


**Figure S2. Morphology of putative mangrove paleosols in supratidal/estuarine facies of the ~50 Ma Delmar formation at Torrey Pines, CA (32.914227, -117.258427).**

Lowermost horizon has abundant root traces up to 2 cm in diameter filled with light-toned fine grained material (possibly detrital kaolinite)


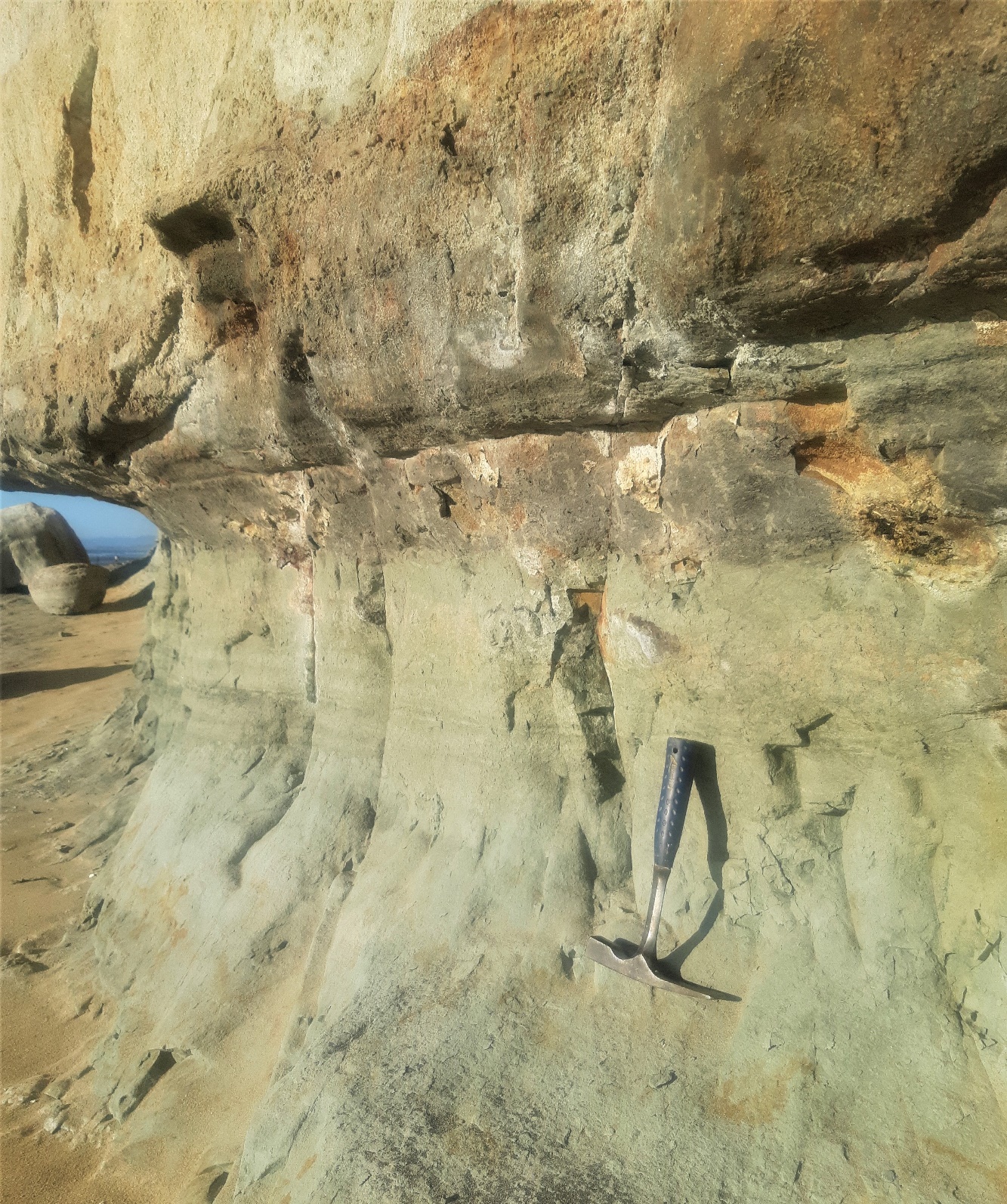
**Figure S3. Field photograph of putative mangrove paleosols in supratidal/estuarine facies of the ~50 Ma Delmar formation at Torrey Pines, CA (upper unit of Figure S2) (32.914227, -117.258427).** Abundant root traces (light streaks) are observed in A-horizon (brown color). Note large (6 cm diameter) coalified fossil wood with yellow-orange jarosite halo in brown A-horizon of upper profile (above right of rock hammer, directly below contact)

**Tables S1-S7 are in the excel file that accompanies this article (Supplementary Data)**

**Table S8. Detailed micromorphological descriptions for Ultisol paleosols at La Jolla, CA**

| **Blacks Beach** | **Voids/Microstructure** | **Coarse-fine distribution** | **Groundmass** | | | | | | |  | **Pedofeatures** | |
| --- | --- | --- | --- | --- | --- | --- | --- | --- | --- | --- | --- | --- |
|  |  |  | **Coarse mineral components** | | | |  | **Micromass** | |  | **coatings** | **nodules** |
|  |  |  | Quartz + feldspar | mica | carbonates | Rock frags. |  | color | b-fabric |  |  |  |
| **S1** | Complex pattern, hard to distinguish from artifacts. No microstructure identified. | Double-spaced porphyric | Predominantly quartz and minor magnetite. Poorly to moderately sorted. Predominantly sub-rounded to sub-angular. Some rounded and angular. Fine to coarse sand. | nd | nd | Sandstone and/or quartzite frags |  | Fe and clay depleted groundmass. Pale brown (light cream) with some intercalations of darker colors. | Weak stipple speckled and striated |  | Infrequent clay coatings associated with planar voids. | nd |
| **S2** | Poorly developed pore network, no structure identified | as above | as above | nd | nd | as above |  | Fe depleted groundmass but not much clay- depleted. | Moderately developed cross and circular striated and granostriated. |  | Clay coatings are more common and seems incorporated in the groundmass. | nd |
| **S3** | Large vesicular and non-accommodating voids | as above | as above | nd | nd | As above |  | Upper part moderately Fe impregnated, lower part is Fe depleted, showing a pale color. | Cross, circular and granostriated. b-fabrics is more intense in the Fe impregnated zone. |  | Common clay coating incorporated in the groundmass.  Common Fe hypocoatings around grains in the Fe depleted zone. | Abundant small to large weak to strongly impregnated orthic Fe nodules. Mainly in the reddish zone. |
| **S4** | Voids look like artifact, but a blocky structure seems likely. | as above | as above | nd | nd | as above |  | Two different colors, a dominant depleted zone with pale colors and a smaller area of light brown color. | Weak birefringence in the pale zone. Stronger striated b-fabric in the not-depleted zone.  Poorly developed stipple speckled. |  | Stronger birefringence along some darker areas, suggest incorporation of clay coatings in the groundmass. Isolated Fe hypocoatings. | Few weakly to strongly impregnated irregular and semi-dendritic Fe-Mn nodules. |
| **S5** | Poorly developed pore network, mostly fine poorly separated. Well accommodating planar voids. | as above | as above | nd | nd | as above |  | Micromass has two different colors, a depleted zone (pale brown) and a not depleted zone (brown). | Cross, circular and granostriated |  | Common Fe hypocoatings. Quartz grains and voids often present a thin Fe coating. Relatively common disturbed clay coatings incorporated in the groundmass. | Few weakly to strongly impregnated irregular and semi-dendritic Fe-Mn nodules. |
| **S6** | as above | as above | as above | Rare biotite | nd | nd |  | There are three zones: a Fe rich, a brownish zone, and a pale brown zone (Fe depleted) | Cross-circular-granostriated |  | Occasional Fe-hypocoating in the depleted zone. Thin Fe coating voids and quartz grains are more common in the brown zone. Relatively common clay coatings incorporated in the groundmass. | A couple of small and moderately Fe impregnation features in the reddish zone. |
| **S7** | Voids seems artifacts | as above | as above | Rare biotite | nd | as above |  | Light brown | Cross-circular-granostriated. Less intense than above. |  | A few Fe hypocoatings and coatings. Relatively common clay coatings but less than above, all incorporated in the groundmass. | nd |
| **nd = not detected** | | | | | | | | | | | | |

**Black's Beach:**

This soil appears to be a deeply weathered, clay-rich Ultisol with abundant redoximorphic features, something like an Aquult. It has an E horizon with evident loss of clay and Fe. Remnants of clay coatings can be detected along some voids, reinforcing the idea of an E horizon. The subsequent horizons are enriched in Fe and clay. Redoximorphic features are common across this profile, indicating poor drainage due to the clay-rich subsurface horizons. The water saturation period seems to have been relatively long, with most of the Fe being precipitated along larger voids, suggesting that microporosity was depleted in oxygen. The initial runiquartz formation suggests strong weathering conditions, which are confirmed by the high CIA values.

Clay coatings in argillic horizons (Bt) are often incorporated into the groundmass, and it can be difficult to recognize them due to the small contrast between illuvial clays and the clayey groundmass and because of pedoturbation. However, it was possible to recognize the clay coatings as they are often associated with voids and show clear extinction lines. They are predominantly limpid and exhibit low interference colors, suggesting kaolinite rather than smectite. In addition to the incorporation of clay coatings in the argillic horizon groundmass, I suggest further incorporation of clay coatings in the groundmass due to post-burial deformation of the clay coatings, as they often appear fragmented and poorly oriented.

This appears to be a continuous profile without evidence of lithological discontinuity. The parent material is uniform across all samples due to similar c/f distribution, mineral composition, roundness, and sorting. Quartz dominates the coarse fraction and predominantly shows a wavy extinction, suggesting a metamorphic origin.

**Interpretation/Comments:**

**S1:** Appears to be an E horizon due to the depletion of Fe and clays.

**Comments:** The presence of remnants of clay coatings along some voids supports the idea of an eluvial horizon.

**S2:** Appears to be the lower part of the E horizon (i.e., E2 or E/Bt horizon).

**Comments:** This sample is similar to the previous one, but this horizon has not been fully clay-depleted and exhibits a stronger b-fabric and more abundant clay coatings.

**S3:** Appears to be a Btg1 horizon. The “g” suffix indicates gleization.

**Comments:** Initial runiquartz formation.

**S4:** Btg2 horizon.

**Comments:** More Fe and clay depleted, indicating longer periods of water saturation.

**S5:** Btg3 horizon.

**Comments:** The more pronounced b-fabric suggests there was no clay depletion like in the above sample, indicating better drainage but still poorly drained. Initial runiquartz formation.

**S6:** Btg4 horizon.

**Comments:** Rare runiquartz formation.

**S7**: Bt horizon or possibly a Bt/C.

**Comments:** Does not appear to be a C horizon due to the strong weathering and clear pedogenic processes (i.e., pedoturbation and clay illuviation). Rare runiquartz formation. The groundmass is more homogeneous, indicating less depletion. I believe the clay-rich horizons above might have promoted poor drainage from rainwater rather than groundwater; otherwise, the lower horizons would have been more Fe and clay depleted. Alternatively, there was not much organic matter at this depth and less oxygen demand.

**Table S3. Detailed micromorphological descriptions for Vertisol paleosols at Cardiff, CA**

| **Cardiff** | **Voids/Microstructure** | **Coarse-fine distribution** | **Groundmass** | | | | | | |  | **Pedofeatures** | |
| --- | --- | --- | --- | --- | --- | --- | --- | --- | --- | --- | --- | --- |
|  |  |  | **Coarse mineral components** | | | |  | **Micromass** | |  | **coatings** | **nodules** |
|  |  |  | Quartz + feldspar | mica | carbonates | Rock frags. |  | color | b-fabric |  |  |  |
| **S30** | Well-developed pore network. Strong and large blocky structure and secondary medium granular. | Open spaced to single spaced porphyric | Quartz, plagioclase. Well to moderately sorted grains, mostly subangular to angular. In some regions, there seems to be infillings of a darker brown, finer material, often associated with larger and more complex pores. Fine to medium sand. | Common biotite, highly weathered | Rare voids filled with carbonates | nd |  | brown | Random striated and granostriated |  | Few clay coatings along some planar voids. Fe hypocoating, and few fine Fe coating | Weakly to moderately impregnated orthic irregular Fe nodules. Common small fragments of Fe nodules dispersed in the groundmass. |
| **S31** | As above but blocky peds are larger and the porosity is less well developed. | Single spaced to double spaced porphyric | as above | as above | as above |  |  | Pale brown  In some regions, there seems to be infillings of a darker brown material associated with larger pores. However, the infilled material has the same texture of the surrounding matrix, which is different from the above sample. | Random striated, and granostriated b fabric |  | Common Fe hypocoating but not coatings. | Weakly to moderately impregnated orthic irregular Fe nodules . Common small fragments of Fe nodules dispersed in the groundmass. |
| **S32** | Poorly developed pore network, when present is usually a thin crack filled with Fe coating-hypocoating | Single spaced porphyric | Quartz. No plagioclase detected. Fine to coarse sand. Moderately to poorly sorted grains, predominantly sub-angular to sub-rounded, some are even rounded. | nd | nd | A few sandstone and/or quartzite fragments |  | 2 tone matrix. A reddish and a depleted matrix. | Randomly to cross-striated and granostriated. |  | Common Fe coatings and hypocoatings in voids in the depleted zone. | A few orthic Fe nodules, aparently more common in the red zone. |
| **S33** | Well developed pore network, typical of vertisols with well developed blocky structure and secondary large/medium granular structure. | Open porphyric | Mostly quartz but with some small plagioclase fragments.  Rare hornblende. Predominantly fine sand and ocasional medium sand | nd | nd | nd |  | Pale and homogeneous matrix but near the more complex voids, the colors are darker, OM? Well to moderately sorted grains. Predominantly sub-rounded and sub-angular. Some angular and some rounded. There seems to be different sources of grains due to differences in roundness but less in size. | Strong randomly striated and less pronounced granostriated b fabrics. |  | nd | nd |
| **S34** | Poorly developed pore network. It has a few large open well accomodated planar voids. Looks like large blocky structure. | Closed porphyric | Mostly quart and common plagioclase and magnetite. Moderately sorted grains. Sub-angular to sub-rounded and some angular and rounded. Fine to medium sized sand. | scattered and frequent mica fragments, strongly weathered | nd | nd |  | Pale and homogeneous matrix. | Random striated and granostriated. |  | nd | A few scatered anorthic small Fe nodules. |
| **S35** | Poorly developed pore network and no evident structure. | as above | Mostly quartz and plagioclase, and magnetite. Moderately sorted grains. Sub-angular to sub-rounded, some angular or rounded. Medium to coarse sand. | as above | Rare carbonate fragment and fillings | nd |  | Pale and homogeneous color. | Stipple speckled. Randomly striated and granostriated. |  | nd | nd |
| **S40** | as above | as above | Mostly quartz and plagioclase, and magnetite. Well sorted grains. Sub-rounded to sub-angular. Fine sand. | as above | nd | nd |  | Groundmass color has three zones. A pale, slightly reddish and a more reddish zone. | Sstipple speckled and moderately developed randomly striated and granostriated. |  | nd | Weakly to moderately impregnated zones of irregular Fe nodules. |
| **S41** | as above | single spaced to closed porphyric. | Mostly quartz and plagioclase, and magnetite. Poorly to moderately sorted grains. Sub-angular to sub-rounded but many angular too.Dominant coarse sand but medium to fine sand. | as above | nd | nd |  | Pale brown to pale. | as above |  | A few and scattered fine Fe coatings along some fine pores | Scattered, not common, strongly impregnated Fe/Mn semi-dendritic nodules |
| **nd = not detected** | | | | | | | | | | | | |

**Interpretation/Comments:**

**Cardiff:** Very dynamic soils with multiple lithologic discontinuities detected (see below). Overall, it looks like a sequence of well-developed Vertisols with moderate to high degree of weathering. The development degree indicates these soils were formed on a stable landscape, but the discontinuities suggest some periods of new material deposition. In the A horizons, the pore size and shape resemble a more grass-type of vegetation, so it might be worth trying to find phytoliths if bulk samples are available. I suspect that some features might be organic, including the darker groundmass in some parts, but it is hard to tell for sure without further analysis.

**S30:** A horizon.

**Comments:** This looks like a typical surficial vertic horizon, highly bioturbated by fine roots, probably grass-like vegetation. Well-developed pore network with incorporation of finer material along cracks.

**S31:** Bss horizon.

**S32:** 2Bss horizon. The “2” prefix indicates a lithologic discontinuity.

**Comment:** Not sure if this is a Bt; it looks more like a vertic horizon (ss suffix) but with a lithological discontinuity due to the sharp differences in grain size and mineralogy with the sample above. The degree of maturity of this sample seems higher too, including more abundant rounded quartz.

**S33:** 3Ab horizon. The “3” prefix indicates another lithologic discontinuity, and the “b” prefix indicates a buried horizon. It looks like an “A” horizon due to the type of porosity that seems related to root activity, likely from grasses.

**Comment:** This is another lithological discontinuity because there is a sharp change in grain size, and plagioclase becomes more common. Also, the well-developed porosity and blocky-granular structure looks more like an A horizon. No iron nodules were detected, suggesting a better-drained environment.

**S34:** 4Bss horizon. The “4” prefix indicates another lithologic discontinuity.

**Comment:** The voids seem to be more the product of pedoturbation, and there is more presence of other primary minerals like plagioclase and biotite.

**S35:** 4Bss2 or a 4BC horizon. It looks like a less developed B horizon than the overlying horizon.

**Comments:** Larger grains and more plagioclase. The b-fabric looks less pedoturbated, with virtually no large pores, and the striation is less visible than the other samples, but it is still strong granostriated. I don't think this is a C horizon because it has been clearly subjected to pedogenesis, maybe a BC horizon.

**S40:** Bssg horizon.

**Comment:** It does not look like a Bt horizon; it looks more like a poorly/moderately developed vertic horizon with relatively poor drainage conditions.

**S41:** 2BC horizon.


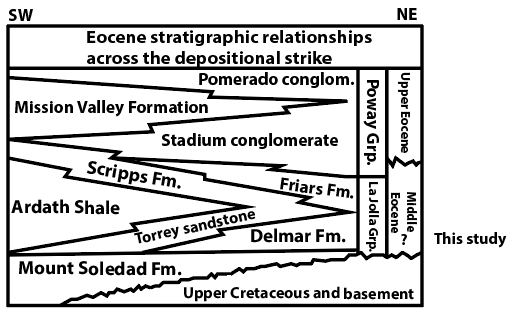


Figure S4. NE to SW oriented stratigraphic section of Eocene stratigraphic relationships across depositional strike in the coastal plain (Adapted from Abbott and May, 1991)

**Abbott, P. L. & Jefferey A. May. Eocene Geologic History: San Diego Region. *SEPM Pacific Sect.* (1991).**
